# A superior extracellular matrix binding motif to enhance the regenerative activity and safety of therapeutic proteins

**DOI:** 10.1038/s41536-023-00297-0

**Published:** 2023-05-22

**Authors:** Yasmin K. Alshoubaki, Yen-Zhen Lu, Julien M. D. Legrand, Rezvan Karami, Mathilde Fossat, Ekaterina Salimova, Ziad Julier, Mikaël M. Martino

**Affiliations:** 1 European Molecular Biology Laboratory Australia, Australian Regenerative Medicine Institute, Monash University, Melbourne, VIC 3800 Australia; 2 Monash Biomedical Imaging, Monash University, Clayton, VIC 3800 Australia; 3 Victorian Heart Institute, Monash University, Clayton, VIC 3800 Australia; 4 Laboratory of Host Defense, World Premier Institute Immunology Frontier Research Center, Osaka University, Osaka, 565-0871 Japan

**Keywords:** Regenerative medicine, Recombinant protein therapy

## Abstract

Among therapeutic proteins, cytokines and growth factors have great potential for regenerative medicine applications. However, these molecules have encountered limited clinical success due to low effectiveness and major safety concerns, highlighting the need to develop better approaches that increase efficacy and safety. Promising approaches leverage how the extracellular matrix (ECM) controls the activity of these molecules during tissue healing. Using a protein motif screening strategy, we discovered that amphiregulin possesses an exceptionally strong binding motif for ECM components. We used this motif to confer the pro-regenerative therapeutics platelet-derived growth factor-BB (PDGF-BB) and interleukin-1 receptor antagonist (IL-1Ra) a very high affinity to the ECM. In mouse models, the approach considerably extended tissue retention of the engineered therapeutics and reduced leakage in the circulation. Prolonged retention and minimal systemic diffusion of engineered PDGF-BB abolished the tumour growth-promoting adverse effect that was observed with wild-type PDGF-BB. Moreover, engineered PDGF-BB was substantially more effective at promoting diabetic wound healing and regeneration after volumetric muscle loss, compared to wild-type PDGF-BB. Finally, while local or systemic delivery of wild-type IL-1Ra showed minor effects, intramyocardial delivery of engineered IL-1Ra enhanced cardiac repair after myocardial infarction by limiting cardiomyocyte death and fibrosis. This engineering strategy highlights the key importance of exploiting interactions between ECM and therapeutic proteins for developing effective and safer regenerative therapies.

## Introduction

Therapeutic proteins have shown significant potential for regenerative medicine applications. For instance, recombinant cytokines and growth factors have been widely explored to promote tissue healing or growth, due to their fundamental roles in the repair and regeneration processes. However, these biologics have often encountered limited clinical success, largely due to low effectiveness, high cost, and major safety concerns^1,2^. For example, a number of pro-regenerative therapeutic protein candidates including recombinant vascular endothelial growth factor-A, interleukin (IL)-10 and many others have failed to progress through the phases of clinical trials, because they were unable to demonstrate significant efficacy^3^. Nevertheless, few cytokines and growth factors have been approved by the US Food and Drug Administration (FDA). For instance, recombinant platelet-derived growth factor (PDGF)-BB has been approved for chronic wound treatment and as a bone stimulating agent for fusion procedures^4,5^. However, despite showing some therapeutic benefits, the use of PDGF-BB has been associated with an increased risk of cancer^4,6^. Another example is recombinant bone morphogenetic proteins (BMPs). These growth factors have been approved as bone stimulating agents^7^, but the use of BMPs may lead to numerous side effects such as hyperinflammation, wound-related complications, spine swelling, and many others^4,8,9^. In addition, the immunogenicity and influence of cytokines and growth factors on the immune system has been recognised as an important concern, with adverse effects such as susceptibility to opportunistic infections, autoimmunity, myelosuppression, and neurological consequences^4^.

High levels of proteolytic activity in injured tissues, often coupled with inflammation, eventually leads to short half-life of locally delivered therapeutic proteins. In addition, recombinant cytokines and growth factors may diffuse rapidly from the site of delivery, making it difficult to achieve therapeutically relevant concentrations^1,10^. Thus, excessive and/or repeated doses of cytokines and growth factors are usually needed to reach a local therapeutic effect. Consequently, in addition to significantly increasing the cost of the treatment, the use of supraphysiological levels of therapeutic proteins clearly augments the probability of triggering significant adverse effects^1,3^. Overall, the limitations that cytokines and growth factors have encountered in the clinic highlight the need to develop better delivery systems that increase their efficacy and safety.

Numerous delivery systems have been explored to control the spatial and temporal delivery of therapeutic proteins^2,10–12^. For cytokines and growth factors, one of the most promising delivery strategies is to mimic how these molecules naturally behave in vivo. Indeed, the activity of cytokines and growth factors is tightly modulated by their interaction with the extracellular matrix (ECM). The ECM protects cytokines and growth factors from enzymatic degradation by proteases, controls their spatial distribution in tissues, and modulates their signalling activity^13,14^. Thus, some interesting approaches rely on conjugating ECM-binding domains to therapeutic proteins of interest, in order to enhance their interaction with endogenous ECM components or ECM-based biomaterial delivery systems^1^. For instance, we found that placenta growth factor (PlGF) binds very strongly to several ubiquitous ECM proteins and heparan sulfate. Thus, we used the ECM-binding motif of PlGF to engineer growth factor and cytokine therapeutics with much higher affinity for ECM proteins compared to their wild-type versions. The engineered proteins were able to significantly improve tissue regeneration at low doses in various animal models^8,15,16^. While the ECM-binding motif from PlGF was demonstrated to be relatively effective, the motif was initially found with a restricted screening among particular growth factor families. Thus, we hypothesised that PlGF was likely not the only protein to have strong and promiscuous binding properties to the ECM. Here, we used a protein motif screening strategy against a protein database coupled with multiple validation approaches to find potentially superior ECM-binding motifs that are suitable for improving the tissue delivery, efficacy, and safety of therapeutic proteins.

In this study, we discovered that the transmembrane protein/growth factor amphiregulin (AREG) possesses an exceptionally strong ECM-binding motif for multiple ubiquitous ECM proteins and heparan sulfate. We used this motif to engineer two clinically approved protein drug models, namely PDGF-BB and IL-1 receptor antagonist (IL-1Ra). We demonstrated that the ECM-binding motif provides extended retention of the therapeutic proteins in tissues when delivered locally. In addition, compared to the ECM-binding motif from PlGF, the ECM-binding motif from AREG significantly reduced the amount of locally delivered therapeutic proteins entering the circulation, which limited tumour growth as a side effect. We ultimately demonstrate that therapeutic proteins engineered with the superior ECM-binding motif from AREG are highly effective at promoting tissue repair or regeneration in three mouse models of injury, including diabetic skin wound healing, muscle regeneration, and cardiac repair following myocardial infarction.

## Results

### Identification of an exceptionally strong ECM-binding motif in AREG

**Fig. 1:**
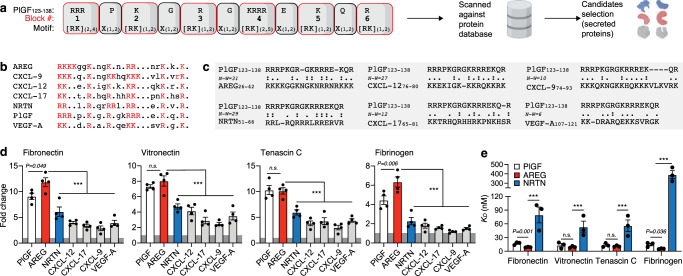
AREG displays exceptionally strong binding to ECM proteins. **a** Sequence motif generated from PlGF_123-138_ used with ScanProsite against the human UniProtKB database to find ECM-binding protein candidates. The 6 lysine and arginine blocks (1-6) are represented in red. The 5 blocks without lysines and arginines are represented in grey. **b** Aligned motif from ECM-binding protein candidates listed in alphabetic order. Blocks of lysine and arginine are in red. **c** Pairwise sequence alignments between PlGF_123-138_ and the motif from protein candidates using the Needleman-Wunsch algorithm. Two dots indicate the same amino acids and one dot indicates amino acids with similar proprieties. Needleman–Wunsch scores (N-W) are shown. **d** Binding of the protein candidates to ECM proteins. ELISA plates were coated with the protein candidates or bovine serum albumin (BSA) control and incubated with ECM proteins. Bound ECM proteins were detected with antibodies. Graphs show fold changes in binding over BSA control. *n* = 4. **e** Binding affinity of PlGF, AREG, and NRTN to ECM proteins. ELISA plates were coated with ECM proteins and incubated with PlGF, AREG, or NRTN at increasing concentrations. Bound PlGF, AREG, and NRTN were detected with antibodies. Graphs show the *K*_*D*_ obtained from the binding curves. *n* = 3. For (**d**, **e**) data are means ± SEM. For (**d**) one-way ANOVA with Bonferroni *post hoc* test for pair-wise comparisons. For (**e**) two-tailed Student’s *t* test. ***P < 0.001, otherwise indicated. n.s., non-significant.

Since the PlGF_123-141_ ECM-binding sequence that we previously discovered has a high number of positively charged amino-acids (Arginine (R) and Lysine (K)), we sought to identify other strong ECM-binding sequences that have this signature. As an approach, we used ScanProsite against the human UniProtKB database, which allows the identification of PROSITE signature matches in protein sequences^17^. We generated a search motif with the following rationale: In the super affinity motif from PlGF, there are six blocks of one to four repeats of R and K residues forming PlGF_123-138_. Given their similarity, R and K are interchangeable and noted “[RK]”. A block of a single repetition of [RK] in PlGF-2_123-138_ can be interpreted as one or two repetitions noted “[RK] (1,2)”. A block of *n* repetitions of [RK] is translated to a block of 2 to *n* + 1 repetitions noted “[RK](2, *n* + 1)” with *n* ≥ 2. Residues separating the blocks of [RK] are translated to one or two repetitions of any residue noted “X (1,2)”. Thus, the search motif generated was [RK]_(2,4)_X_(1,2)_[RK]_(1,2)_X_(1,2)_[RK]_(1,2)_X_(1,2)_[RK]_(2,5)_X_(1,2)_[RK]_(1,2)_X_(1,2)_[RK]_(1,2)_ (Fig. 1a).

Given that PlGF is a growth factor, we focused the search on secreted proteins where the ECM-binding property is likely to have been an evolutionarily selected function, such as the one found in cytokines and growth factors. Since cytokines and growth factors are usually between 100 and 400 amino acids, we restricted the search to proteins with a maximum of 500 amino acids. The search gave 248 hits in 191 sequences (Supplementary Table 1). A large fraction of the protein hits were intracellular proteins and thus not selected. Then, the remaining proteins were filtered to keep only known cytokines and growth factors. Based on the search strategy, six protein candidates were found, namely AREG, (C-X-C motif) ligand (CXCL)-9, CXCL-12, CXCL-17, neurturin (NRTN), and VEGF-A (Fig. 1b). In addition, we used pairwise sequence alignment using the Needleman-Wunsch algorithm to find an optimal alignment between PlGF_123-138_ and the sequences that were found (Fig. 1c). Alignment scores (Needleman-Wunsch score) showed that AREG and NRTN both contained a sequence that aligned the most to PlGF_123-138_. Local alignment using the Smith-Waterman algorithm and Waterman-Eggert score showed the same trend (Supplementary Fig. 1).

To validate the search results and assess whether the protein candidates display high affinity to ECM proteins, we used an approach based on enzyme-linked immunosorbent assay (ELISA). Binding was tested on fibronectin, vitronectin, tenascin C and fibrinogen, which are ubiquitous ECM proteins known to display growth factor and cytokine binding abilities^15,18–21^. Interestingly, although the top candidates AREG and NRTN showed binding properties, AREG binding to ECM proteins was much stronger and significantly higher than PlGF for fibronectin and fibrinogen (Fig. 1d). Next, we measured the binding affinity of PlGF, AREG, and NRTN to the ECM proteins. AREG showed very strong binding to all ECM proteins with dissociation constants (*K*_*D*_) in the nM range, while NRTN showed significantly less binding capacity. Remarkably, the affinity of AREG for fibronectin and fibrinogen was significantly higher compared to PlGF (Fig. 1d, Supplementary Table 2, Supplementary Fig. 2).

**Fig. 2:**
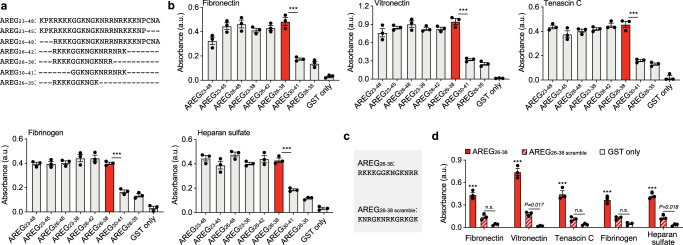
AREG_26-38_ is the minimal ECM-binding sequence from AREG. **a** AREG fragment sequences fused to GST. **b** Binding of AREG fragments to ECM proteins. ELISA plates were coated with ECM proteins and incubated with AREG fragments or GST only. Graphs show signals given when detecting GST. AREG_26-38_ is shown in red. *n* = 3. **c** AREG_26-38_ and AREG_26-38_ scramble sequences fused to GST. **d** Binding of AREG_26-38_ and AREG_26-38_ scramble to ECM proteins. ELISA plates were coated with ECM proteins and incubated with AREG_26-38_, AREG_26-38_ scramble, or GST only. Graphs show signals given when detecting GST. *n* = 3. For (**b**, **d**) data are means ± SEM. One-way ANOVA with Bonferroni *post hoc* test for pair-wise comparisons. ***P < 0.001, otherwise indicated; n.s., non-significant.

Then, to determine the minimal AREG sequence that still allows strong binding to ECM, we generated seven AREG fragments overlapping the sequence that was found with ScanProsite (Fig. 2a). Sequences were fused to glutathione S-transferase (GST) and binding to ECM proteins and heparan sulfate was tested. Interestingly, the smallest AREG sequence that was able to bind ECM protein and heparan sulfate was a relatively small sequence of 13 amino acids, AREG_26-38_ (Fig. 2b). In addition, we tested the binding of AREG_26-38_ to collagen I and II, since collagens are highly abundant in the ECM. AREG_26-38_ showed binding to both collagens (Supplementary Fig. 3). As the sequence is highly positively charged, we tested whether the overall charge or actual order of the amino acids was critical for binding. Remarkably, a scrambled AREG_26-38_ with the same charge showed poor binding to ECM proteins and heparan sulfate, compared to AREG_26-38_, suggesting that the positions of the amino acids in the sequence are critical (Fig. 2c, d). Overall, we found that AREG displays exceptionally strong binding to ECM proteins via AREG_26-38_.

### Therapeutic proteins engineered with the ECM-binding motif from AREG display high retention in tissues with limited side effects

We thought to use the ECM-binding motif of AREG to improve protein drug retention, activity, and safety following delivery. As therapeutic protein models, we selected PDGF-BB and interleukin-1 receptor antagonist (IL-1Ra). PDGF-BB is a key growth factor with regenerative activities in multiple tissues but has raised safety concerns regarding its potential tumorigenic effect when delivered at therapeutic doses and efficacy issues when delivered at lower doses^4,6^. IL-1Ra is also a very promising therapeutic for regenerative medicine applications but the antagonist is usually used at exceedingly high doses with multiple administrations and its usage can lead to side effects such as immune reactions and infections^22,23^.

**Fig. 3:**
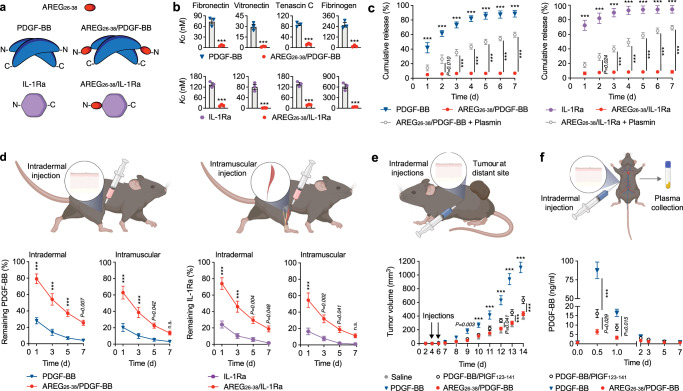
Therapeutic proteins fused to AREG_26-38_ display high retention in tissues with limited off-target effects. **a** Fusion strategy for PDGF-BB (in blue) and IL-1Ra (in purple). AREG_26-38_ is represented in red, and N and C indicate the N- and C-terminus, respectively. **b** Binding affinity of PDGF-BB and IL-1Ra to ECM proteins. ELISA plates were coated with ECM proteins and incubated with PlGF, AREG, or NRTN at increasing concentrations. Bound PlGF, AREG, and NRTN were detected with antibodies. Graphs show the binding affinity (*K*_*D*_) obtained from the binding curves. *n* = 3. **c** Release kinetics of PDGF-BB and IL-1Ra variants from an ECM-mimetic hydrogel. PDGF-BB or IL-1Ra variants were incorporated in ECM-mimetic hydrogels constituted of fibronectin, vitronectin, tenascin C, fibrinogen and heparan sulfate. Hydrogels were incubated in 10 times volume of buffer, with or without plasmin, that was harvested and replaced every 24 h. Graphs show the cumulative release of PDGF-BB and IL-1Ra variants in buffer. *n* = 4 per time point. **d** Retention of PDGF-BB and IL-1Ra following delivery in tissues. IL-1Ra and PDGF-BB variants were injected in mouse dorsal skin or the tibialis anterior muscle. Graphs show the percentage of PDGF-BB and IL-1Ra variants remaining at the site of injection at various time points. *n* = 4 per time point. **e** Tumour growth following delivery of PDGF-BB variants at a distal site. Tumour cells were injected subcutaneously. PDGF-BB variants or saline control were injected intradermally 2 cm further at 4 and 6 days after tumour implantation. The graph shows the growth of the tumour over 14 days. *n* = 6. **f** Serum concentration of PDGF-BB variants following intradermal delivery. PDGF-BB variant concentrations were measured at different time points after injection. *n* = 4 per time point. In (**b**–**f**) data are means ± SEM. For (**b**) two-tailed Student’s *t* test. For (**c**–**f**) two-way ANOVA with Bonferroni *post hoc* test for pair-wise comparisons. In (**e**) comparisons are between AREG_26-38_ and the other groups. ***P < 0.001, otherwise indicated. n.s., non-significant.

AREG_26-38_ forms part of the N-terminus of AREG. Therefore, we engineered PDGF-BB (a homodimer) and IL-1Ra (a monomer) with an N-terminus fusion of AREG_26-38_ to mimic the natural position of the motif in AREG (Fig. 3a). Fusion of AREG_26-38_ to PDGF-BB and IL-1Ra did not disrupt their biological activity, as shown by the ability of AREG_26-38_/PDGF-BB to stimulate dermal fibroblast proliferation and AREG_26-38_/IL-1Ra to inhibit the release of IL-6 by macrophages when stimulated with IL-1 (Supplementary Fig. 4). However, fusion of AREG_26-38_ resulted in a drastic enhancement of binding to ECM proteins ranging from a 5- to 50-fold increase in binding affinity (Fig. 3b, Supplementary Table 3, Supplementary Fig. 5). Then, we tested the ability of engineered PDGF-BB and IL-1Ra to stay sequestered in the ECM utilising an ECM-mimetic hydrogel composed of common ECM components. The ECM-mimetic hydrogel was based on a fibrin matrix containing fibronectin, vitronectin, tenascin C, and heparan sulfate. All these ECM components were incorporated in the hydrogel via protein-protein interactions, following fibrin polymerisation initiated by the thrombin-induced proteolytic conversion of fibrinogen^16^. Fibronectin is crosslinked to fibrin through the transglutaminase factor XIIIa (present in fibrinogen preparations). Vitronectin directly interacts with fibrin, and tenascin C binds fibronectin. Fibrin, fibronectin, vitronectin, and tenascin C all have heparin-binding domains capturing heparan sulfate in the hydrogel. AREG_26-38_/PDGF-BB and AREG_26-38_/IL-1Ra were retained in the ECM-mimetic hydrogel while the wild-type forms were quickly released. In addition, AREG_26-38_/PDGF-BB and AREG_26-38_/IL-1Ra were gradually released in the presence of plasmin, a protease that is present in injured tissues and cleaves the ECM proteins of the hydrogel^15^ (Fig. 3c). Interestingly, we also found that AREG_26-38_ can be cleaved by plasmin, due to its multiple potential plasmin cleavage sites (Supplementary Fig. 6). To further validate this very strong ECM-binding capacity in vivo, we then tested the retention of the engineered protein therapeutics after intradermal and intramuscular administration in mice. As predicted, PDGF-BB and IL-1Ra engineered with AREG_26-38_ showed extended retention in both skin and muscle tissues with about 10-20% of the initial dose still present at the delivery site after a week, while most of the wild-type proteins diffused away from the delivery site within three days (Fig. 3d). Then, we tested the retention of AREG_26-38_-fused therapeutics in the context of tissue injury. IL-1Ra variants were delivered intradermally at the border of full-thickness wounds and PDGF-BB variants were delivered via fibrin in volumetric muscle loss injuries. The AREG_26-38_-fused therapeutics showed extended retention at the delivery site in both injury models (Supplementary Fig. 7a). To explore the pharmacokinetics of the engineered therapeutics further, we measured their concentrations in kidney, liver, lung, and spleen over seven days. Correlating with the strong local retention, AREG_26-38_/IL-1Ra and AREG_26-38_/PDGF-BB were barely detected in all tissues, while significantly higher concentrations of wild-type PDGF-BB and IL-1Ra were detected after 24 hours (Supplementary Fig. 7b).

We next explored if high local retention in tissues can limit off-target effects. As a model, we used the acceleration of tumour growth induced by PDGF-BB. Tumour cells were implanted subcutaneously in the back of mice and high doses of PDGF-BB variants were then injected intradermally at a distal site from the tumour cells four- and six-days following cell implantation. Compared to saline, injection of PDGF-BB resulted in significantly faster tumour growth from day 9, while delivery of AREG_26-38_/PDGF-BB did not significantly enhance tumour growth (Fig. 3e). Interestingly, PDGF-BB/PlGF_123-141_ resulted in a small enhancement of tumour growth, which was significantly higher compared to AREG_26-38_/PDGF-BB (Fig. 3e). To understand if the enhancement of tumour growth was directly correlated to a higher concentration of circulating PDGF-BB, we measured the concentration of PDGF-BB in plasma at various time points (up to seven days) following intradermal delivery. As expected, we could detect significant amounts of wild-type PDGF-BB during the first 24 h post-injection, which rapidly decreased over time. Remarkably, the concentration of AREG_26-38_/PDGF-BB was approximately ten times lower compared to wild-type PDGF-BB and quickly decreased during the following days. In addition, AREG_26-38_/PDGF-BB concentration was significantly lower than PDGF-BB/PlGF_123-141_, suggesting that AREG_26-38_/PDGF-BB may trigger fewer off-target effects (Fig. 3f).

### PDGF-BB engineered with the ECM-binding motif from AREG promotes diabetic skin wound healing and muscle regeneration

**Fig. 4:**
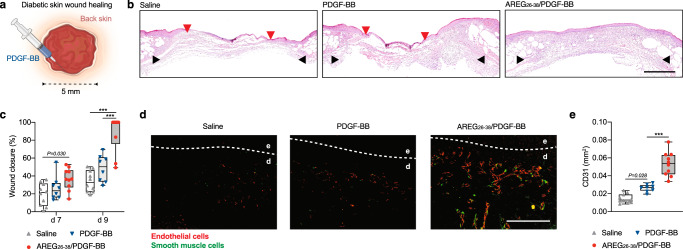
AREG_26-38_/PDGF-BB accelerates diabetic skin wound healing. **a** Full-thickness wounds (5 mm diameter) in diabetic mice (*Lepr*^*db/db*^) were treated with PDGF-BB variants (0.5 μg of wild-type, equimolar AREG_26-38_/PDGF-BB). *n* = 10 for saline and AREG_26-38_/PDGF-BB, *n* = 8 for PDGF-BB. **b** Representative histology (haematoxylin and eosin staining) 9 days post-treatment. Black arrows indicate wound edges and red arrows indicate tips of epithelium tongue. The epithelium (if any) appears in purple as a homogeneous keratinocyte layer on top of the wounds. The granulation tissue under the epithelium contains granulocytes with dark-purple nuclei. Fat tissue appears as transparent bubbles. Scale bar, 1 mm. **c** Wound closure evaluated by histomorphometric analysis of tissue sections. **d**, **e** Angiogenesis at day 9 assessed by immunostaining of wound sections for CD31 (endothelial cells, red) and desmin (smooth muscle cells, green). Representative images in **d**. Dashed lines indicate separation between epidermis (indicated as e) and dermis (indicated as d). Scale bar, 0.2 mm. Quantification of CD31 in **e**. In (**c,** **e**) data are plotted in box plots, box shows the median (centre line) and IQR (bounds). Whiskers show the minimum/maximum range. Two-way ANOVA with Bonferroni *post hoc* test for pair-wise comparisons for (**c**). One-way ANOVA with Bonferroni *post hoc* test for pair-wise comparisons for (**e**). ***P < 0.001, otherwise indicated.

As PDGF-BB is approved for the treatment of diabetic ulcers^4,5^, we explored whether low doses of AREG_26-38_/PDGF-BB could promote wound closure in a model of impaired wound healing in diabetic mice. Full-thickness wounds were generated on the back skin and treated with a single low dose of PDGF-BB variants (0.5 μg of PDGF-BB or equimolar of AREG_26-38_/PDGF-BB) or saline controls (Fig. 4a). Wild-type PDGF-BB treatment did not significantly improve wound closure compared to saline. However, AREG_26-38_/PDGF-BB significantly accelerated wound closure with most of the wounds closed with a layer of epidermis nine days following treatment. In addition, wound closure with AREG_26-38_/PDGF-BB was significantly higher compared to wild-type PDGF-BB (Fig. 4b, c). Since angiogenesis is critical for wound closure, we assessed the extent of new blood vessel formation in the granulation tissue following treatments. Compared to saline and wild-type PDGF-BB, AREG_26-38_/PDGF-BB delivery resulted in the formation of more blood vessel structures comprising endothelial cells and to some extent smooth muscle cells (Fig. 4d, e). Overall, treatment of diabetic wounds with a low dose of AREG_26-38_/PDGF-BB promoted rapid wound closure.

**Fig. 5:**
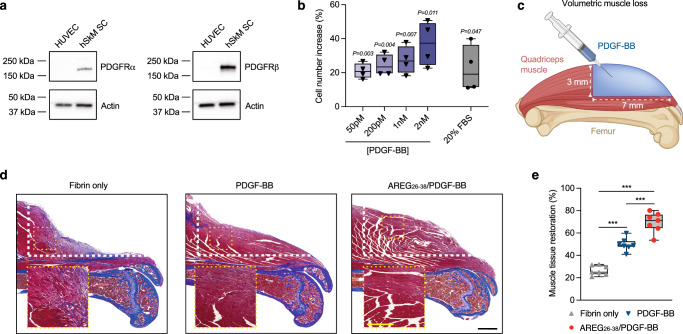
AREG_26-38_/PDGF-BB promotes muscle regeneration. **a** PDGFRα and PDGFRβ expression in primary human muscle satellite cells (hSkM SCs) and human umbilical vein endothelial cells (HUVECs) as negative control cells. The western blots show expression of PDGFRα, PDGFRβ and Actin control. **b** hSkM SCs proliferation in response to PDGF-BB. Cells were stimulated for 2 days and the increase in cell number compared to saline treatment was measured. Foetal bovine serum (FBS) was used as a positive control. *n* = 4. **c** A volumetric muscle loss defect was created in the quadriceps of wild-type mice and treated with a fibrin hydrogel only or fibrin with PDGF-BB variants (2 μg of wild-type, equimolar AREG_26-38_/PDGF-BB). After 3 weeks, the level and quality of muscle tissue regenerated was assessed by histology. **d** Representative histology sections of the centre of the muscle defect stained with Masson’s trichrome. White dashed lines indicate where muscle tissue was injured. Yellow boxes indicated magnified areas of the regenerated muscle. Black scale bar, 1 mm; yellow scale bar, 500 μm. Muscle tissue is stained in dark red, fibrotic parts are in blue/violet, and the femur stained in blue appears under the muscle. **e** Quantification of muscle tissue restoration at the centre of the defect expressed as a percentage muscle area of the uninjured right leg. *n* = 7. In (**a**, **e**) data are plotted in box plots, box shows the median (centre line) and IQR (bounds). Whiskers show the minimum/maximum range. One-sample *t* test with hypothetical value of 0 in (**a**). One-way ANOVA with Bonferroni *post hoc* test for pair-wise comparisons in (**e**). ***P < 0.001, otherwise indicated.

While the role of PDGF-BB during muscle regeneration is still elusive, recent reports suggest that PDGF receptor (PDGFR) signalling is critical for muscle regeneration^24^. We found that in vitro cultured primary human skeletal muscle stem cells express both PDGFRα and PDGFRβ, and their ligand PDGF-BB stimulated their proliferation (Fig. 5a, b). Therefore, we explored if local delivery of PDGF-BB could promote muscle regeneration. As an approach, we used a volumetric muscle loss model in mice that we recently established^25^. In this model, a large proportion of the quadriceps muscle group is removed, and the defect cannot fully regenerate without therapeutic intervention (Fig. 5c, Supplementary Fig. 8). Defects were treated with PDGF-BB or AREG_26-38_/PDGF-BB (2 μg of PDGF-BB or equimolar of AREG_26-38_/PDGF-BB) delivered via a fibrin matrix directly polymerised in the defect. The fibrin matrix alone was used as a control, and muscle regeneration was assessed by histology three weeks after treatment. Interestingly, PDGF-BB was able to significantly regenerate part of the muscle defect compared to fibrin only. However, AREG_26-38_/PDGF-BB showed a much better ability to regenerate muscle compared to wild-type PDGF-BB (Fig. 5d, e). Taken together, we demonstrated that AREG_26-38_/PDGF-BB can promote regeneration of large muscle defects.

### IL-1Ra engineered with the ECM-binding motif from AREG improves repair outcome after myocardial infarction

**Fig. 6:**
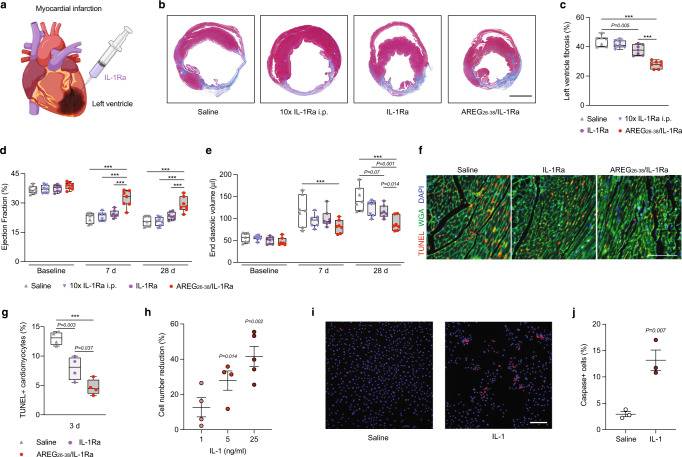
Intramyocardial delivery of AREG_26-38_/IL-1Ra following myocardial infarction limits fibrosis and improves healing. **a** Permanent left coronary artery ligation was performed in mice to create a myocardial infarction. IL-1Ra was delivered intraperitoneally (40 μg) on the day of surgery, or the borders of the infarct regions were injected with saline or IL-1Ra variants (4 μg of wild-type, equimolar AREG_26-38_/IL-1Ra). *n* = 7. **b** Representative histology (Masson’s trichrome) 28 d post-treatment. Healthy cardiac tissue appears in red and fibrotic tissue appears in blue/purple. Scale bar, 2 mm. **c** Histological quantification of fibrosis. **d**, **e** Ejection fraction and end-diastolic volume quantified by echocardiogram analysis at baseline, day 7 and 28 post-infarction. **f** Representative images of immunostaining detecting apoptotic cardiomyocytes 3 days post-infarction. Nuclei are in blue, wheat germ agglutinin (WGA) labels cell membrane in green, TUNEL positive staining indicative of apoptotic cells in red. Scale bar, 100 μm. **g** Quantification of apoptotic cardiomyocytes. **h**–**j** Cardiomyocyte death induced by IL-1. Human cardiomyocytes were treated with IL-1 for 72 h. **h** The graph shows the reduction of cell number compared to saline treatment (*n* = 4 for 1 and 5 ng/ml, *n* = 5 for 25 ng/ml). **i**, **j** Pan-caspase activity was detected in cardiomyocytes treated with IL-1 (25 ng/ml, *n* = 3) and representative immunostainings are shown. Scale bar, 500 μm. **j** Quantification is shown. In (**c**–**e**, **g**) data are plotted in box plots, box shows the median (centre line) and IQR (bounds). Whiskers show the minimum/maximum range. In (**h**, **j**) data are means ± SEM. One-way ANOVA with Bonferroni *post hoc* test for pair-wise comparisons in (**c**, **g**). Two-way ANOVA with Bonferroni *post hoc* test for pair-wise comparisons in (**d**, **e**). One-sample *t* test with hypothetical value of 0 in (**h**). Student’s *t* test in (**j**). ***P < 0.001, otherwise indicated.

IL-1 receptor signalling in the heart following myocardial infarction has been shown to be detrimental for cardiac repair, as it leads to an increase in fibrosis and long-term impairment of heart function^26^. Thus, inhibition of IL-1 receptor signalling is an attractive strategy to improve healing outcome after myocardial infarction^27–29^. Indeed, Phase II clinical trials in which a high dose of IL-1Ra was delivered systemically for several days showed a lower incidence of death in patients with acute myocardial infarction or heart failure with preserved ejection fraction^30–34^. Thus, we explored if systemic delivery of IL-1Ra or an intramyocardial local delivery of a low dose of IL-1Ra with or without very strong affinity for the ECM could lead to improved cardiac repair after myocardial infarction in mice. For local delivery, IL-1Ra variants (1 μg of IL-1Ra or equimolar of AREG_26-38_/IL-1Ra) or saline control were injected in four sites surrounding the infarct area following permanent left coronary artery ligation (Fig. 6a). For systemic delivery, a ten times higher dose of IL-1Ra (40 μg) was delivered intraperitoneally on the day of surgery. One week after treatment, mice that received systemic delivery of wild-type IL-1Ra did not display improved cardiac function. Local delivery of wild-type IL-1Ra had a slight but not significant improvement in cardiac repair, in comparison to the saline group. On the other hand, local delivery of AREG_26-38_/IL-1Ra significantly improved cardiac repair outcome and function compared to IL-1Ra, as evidenced by the improved ejection fraction of the left ventricle and reduced fibrosis, reduced end-diastolic volume, improved end-systolic anterior wall thickness, and reduced internal diameter of the left ventricle (Fig. 6b–e, Supplementary Fig. 9 and 10, Supplementary Video 1–4). The positive effect in cardiac repair outcomes was equally conserved four weeks post-myocardial infarction.

To gain insight into the mechanisms by which IL-1Ra variants facilitate cardiac repair, we measured cardiomyocyte apoptosis three days after treatment, as cardiomyocyte death is one of the main events leading to fibrotic remodelling after myocardial infarction^35^. Local delivery of wild-type IL-1Ra reduced apoptotic cardiomyocyte cell death in the infarct zone of the left ventricle, but AREG_26-38_/IL-1Ra was significantly better at reducing apoptosis, compared to wild-type IL-1Ra (Fig. 6f, g). We then explored whether IL-1 could directly trigger cardiomyocyte death. As a model, we used induced pluripotent stem cell (iPSC)-derived human cardiomyocytes. Stimulation of cardiomyocytes with IL-1 led to cell death in a dose- dependent manner (Fig. 6h). In addition, we found that IL-1 induced significant pan-caspase activity in cardiomyocytes (Fig. 6i, j), suggesting that IL-1 triggers cardiomyocyte death directly. Overall, these results indicated that local delivery of IL-1Ra improved repair outcome after myocardial infarction to some extent, but IL-1Ra engineered with the ECM-binding motif from AREG displayed vastly superior effectiveness.

## Discussion

Despite their evident therapeutic potential for regenerative medicine, therapeutic proteins such as recombinant cytokines and growth factors have encountered significant setbacks that have impeded their widespread adoption in the clinic^1,2^. To resolve the limitations of cytokines and growth factors as drugs and increase their efficacy and safety, it is critical to develop optimal delivery systems that precisely control their localisation and signalling following delivery. Given the ECM closely controls cytokine and growth factor activities, delivery systems based on protein engineering approaches that aim to modulate interactions with the endogenous ECM in the target tissue or ECM-based biomaterials have shown encouraging results in pre-clinical models^8,15,16,21,36,37^. Another benefit of this approach is that the engineered therapeutic protein contains a “built in” delivery system and therefore does not require the use of complex biomaterials or in some cases a biomaterial at all. Consequently, a less convoluted therapeutic approach might facilitate the regulatory path for approval. Particularly, it has been demonstrated that engineering therapeutic proteins with the ECM-binding motif from PlGF increases the efficacy of the drug at low doses not only in the context of regenerative medicine applications^8,15,16^, but also in other settings such as cancer immunotherapy^38,39^. Nevertheless, we anticipated that other and potentially superior ECM-binding motifs likely exist within the human proteome. The advantage of searching for an ECM-binding motif derived from a human protein rather than generating a purely synthetic motif is the reduced risk of developing immunogenicity for a neo-epitope.

Since ECM-binding domains commonly have a high number of positively charged amino acids, the rationale for generating the search motif with ScanProsite relied mainly on the pattern of positively charged amino acid blocks found in PlGF_123-138_. While about 200 protein hits were found, many of the proteins were nuclear and DNA-binding proteins. This result was anticipated, due to the positively charged nature of the motif, as DNA-binding proteins usually have positively charged sequences. We decided to disregard intracellular proteins and proteins that were not known cytokines or growth factors to identify candidates where the ECM-binding property is likely linked to their regulation and biological function. Interestingly, among the six candidates that possessed a similar ECM-binding motif to PlGF, none were found using BLAST with PlGF_123-138_ as a sequence query. Among the six protein candidates we found to have a similar ECM-binding motif to PlGF, the motif in AREG showed the best alignment scores with PlGF_123-138_. In line with the alignment scores, the ECM binding assays further confirmed that AREG has a much higher binding affinity to ECM components compared to the other protein candidates. Indeed, AREG has been reported to bind heparan sulfates in the ECM, which is a property that likely modulates its morphogenic activity^40^. However, no reports have previously shown direct binding to specific ECM proteins. In addition, we found that the affinity of AREG for fibronectin and fibrinogen was significantly higher compared to PlGF, suggesting that the AREG motif may have increased capacity to bind the ECM in vivo. This assumption is supported by the significantly lower concentration of AREG_26-38_/PDGF-BB detected in the circulation compared to PDGF-BB/PlGF_123-141_ following intradermal injection.

As therapeutic protein models, we selected PDGF-BB and IL-1Ra, because both proteins are promising for multiple applications and are clinically approved but face important obstacles that limit their widespread adoption. One of the main issues with PDGF-BB is that its use has been associated with an increased risk of cancer and recombinant PDGF-BB (becaplermin, Regranex) has a black box warning issued by USFDA^4,6^. Utilising a tumour model in the mouse, we showed that tumour growth is accelerated when PDGF-BB is delivered at a site distant to the tumour. However, we demonstrated that engineering PDGF-BB with the AREG ECM-binding motif abolished this adverse effect. Interestingly, PDGF-BB/PlGF_123-141_ still had a slight tumour growth effect. This result reiterates that the ECM-binding motif from AREG may be more effective than that of PlGF. On the other hand, the use of recombinant IL-1Ra (anakinra, Kineret), which is administered systemically for different applications has been linked with adverse effects such as immunogenicity and infections^22,23^. The adverse effects likely come from the need for daily subcutaneous injections of an extremely high dose (100 mg) due to the short half-life of IL-1Ra^4,41^. In addition, the systemic delivery of IL-1Ra and immunomodulators in general is more likely to trigger immune-related adverse side effects^4,42^. Here, we demonstrated that therapeutic proteins engineered with the ECM-binding motif from AREG were effectively retained locally following delivery in non-injured and injured tissues, while non-modified proteins quickly diffused away from the delivery site. The high tissue retention of therapeutic proteins engineered with AREG_26-38_ was likely due to the robust interaction with ECM components that prevented their diffusion away from delivery sites. In addition, binding of the therapeutic proteins to the ECM could provide better protection against extracellular proteases, which is a well described mechanism^43^. Although we demonstrated that the AREG motif is cleaved by plasmin, it is likely that plasmin is not the only mechanism by which the engineered therapeutics are released from ECM. Indeed, the ECM undergoes turnover and is significantly remodelled during tissue repair and regeneration by the activity of several enzymes such as matrix metalloproteinases (MMPs)^44^. Thus, release of the therapeutics will also depend on ECM remodelling during the healing process. In addition, it is possible that some MMPs have the capacity to cleave the AREG motif. Lastly, while ECM composition may vary significantly between tissues and is also influenced by other factors such as aging and inflammation, the ECM-binding motif in AREG showed promiscuous binding to a range of ubiquitous ECM proteins and heparan sulfate. Thus, the protein engineering strategy based on this motif capitalises on its versatility, which allows application in various tissues.

Recombinant PDGF-BB is recognised as one of the most promising growth factor therapeutics to promote healing of chronic wounds^45^. Thus, as a first tissue repair model, we tested the potential of engineered PDGF-BB to promote skin wound healing in diabetic mice. The *Lepr*^*db/db*^ mouse is widely used for pre-clinical evaluation of wound healing therapeutics, as this model of impaired wound healing mimics some aspects of human chronic wounds^46^. Since AREG_26-38_/PDGF-BB showed high tissue retention following delivery in skin, we selected a relatively low dose that was approximately ten times lower than what has been reported to be effective with wild-type PDGF-BB in similar models^47,48^. Remarkably, we found that a low dose of AREG_26-38_/PDGF-BB delivered topically was able to close most of the wounds nine days after delivery, while wild-type PDGF-BB was largely ineffective. This was somewhat expected given the low dose delivered of the growth factor and the lack of delivery system. There are likely multiple mechanisms by which AREG_26-38_/PDGF-BB promoted wound healing in diabetic mice. For instance, PDGF-BB is known to regulate the proliferation and migration of various mesenchymal lineage cell types that are important for wound healing^13^. In addition, PDGF-BB is well-known to have a critical role in angiogenesis and stabilising new blood vessels^13,49^. Indeed, we found that angiogenesis in the granulation tissue was much more developed in AREG_26-38_/PDGF-BB-treated wounds.

As a second tissue repair model, we used a volumetric muscle loss model in mice to assess engineered PDGF-BB. Volumetric muscle loss is a significant clinical problem that is often related to traumatic injuries from accidents or battlefields but is also a consequence of certain musculoskeletal surgeries such as hip replacement^50,51^. Although many regenerative strategies based on biomaterials and therapeutic proteins have been explored^52,53^, there are currently no approved therapeutics for muscle reconstruction. Several strategies based on the delivery of growth factors such as VEGF-A and insulin-like growth factor have shown encouraging results in pre-clinical models^54,55^. However, the potential of delivering PDGF-BB for regenerating volumetric muscle loss was largely unknown. Thus, considering that AREG_26-38_/PDGF-BB showed very high retention after delivery in muscle via fibrin, we selected a dose that was approximately five times lower than what has been reported with other volumetric muscle loss models in mice^54,55^. Strikingly, we found that AREG_26-38_/PDGF-BB is effective at stimulating muscle regeneration, showing substantially more regenerative activity compared to wild-type PDGF-BB. Histological analysis also revealed that the fibrin matrix was degraded after three weeks and the regenerated tissue treated with AREG_26-38_/PDGF-BB was mostly muscle without fibrosis. Large fibrotic areas were not detected, indicating that the newly formed muscle with AREG_26-38_/PDGF-BB was likely functional, although further analysis such as strength experiments at later time points would be required to confirm to what extent muscle function is recovered. AREG_26-38_/PDGF-BB likely stimulated muscle regeneration through multiple mechanisms. Firstly, it is well known that proliferation of muscle satellite cells is central for muscle regeneration, and we found that PDGF-BB can stimulate human muscle satellite cells in a dose-dependent manner probably via PDGFRα and/or PDGFRβ. Secondly, it has been shown that PDGF receptor α (PDGFRα) in skeletal muscle-resident fibro-adipogenic progenitors is critical during muscle regeneration^24^. Lastly, AREG_26-38_/PDGF-BB may have a positive effect on angiogenesis similar to what we observed in the diabetic wound healing model.

Finally, we used the myocardial infarction model in the mouse to test the efficacy of engineered IL-1Ra. Indeed, one of the most promising applications of IL-1Ra in regenerative medicine is to limit damage after myocardial infarction, as IL-1 receptor signalling is a major negative regulator of cardiac repair^26^. Mechanistically, it has been shown that necrotic cells resulting from ischaemia release danger signals that activate the inflammasome in cardiomyocytes, fibroblasts, immune cells, and vascular cells. Activation of the inflammasome leads to the release of IL-1β^56–59^, which amplifies the inflammatory response that further exacerbates cardiomyocyte death by activation of caspases and via other mechanisms^27,35,60,61^. Thus, inhibition of IL-1 receptor signalling after myocardial infarction could be a very effective approach to promote cardiac repair. Multiple clinical trials have shown encouraging results with the use of IL-1Ra. However, these results were achieved by systemic delivery of IL-1Ra every day at a very high dose (100 mg per injection), which is not a cost-effective method and can lead to adverse effects^32,62–64^. Thus, we tested whether local delivery of a low dose of AREG_26-38_/IL-1Ra directly in the ventricle wall would be effective. The dose was selected based on other mouse models where an ECM-binding form of IL-1Ra has been shown to be effective at inhibiting IL-1 signalling^8,16^. Intramyocardial delivery of biologics in clinical settings can be achieved using catheter systems to accurately inject therapeutics in the left ventricle^65,66^. In our murine model, we used a microinjection syringe system to mimic this practice. Remarkably, treatment with AREG_26-38_/IL-1Ra was very effective at promoting cardiac repair as it significantly reduced left ventricular fibrosis and improved overall cardiac function following myocardial infarction, compared to wild-type IL-1Ra and systemic delivery of a high dose of IL-1Ra. Giving insights about the mechanisms of action, we showed that the delivery of AREG_26-38_/IL-1Ra significantly reduced cardiomyocyte death after myocardial infarction, which is critical to limit subsequent cardiac damage and remodelling. In addition, we found that treatment of human cardiomyocytes with IL-1 induced caspase activation and cell death in a dose-dependent manner. Thus, these findings suggest that AREG_26-38_/IL-1Ra directly prevents cardiomyocyte death in vivo. Nevertheless, there are likely other mechanisms by which IL-1 receptor inhibition improves the cardiac function, such as regulating immune cell functions and the inflammatory response of other non-cardiomyocyte cells in the heart that express the IL-1 receptor^16,29^.

In conclusion, we highlight the key importance of leveraging interactions between the ECM and protein therapeutics for developing effective and safer regenerative therapies. In the context of regenerative medicine, we demonstrated that engineering therapeutic proteins with a superior ECM-binding motif derived from AREG considerably increases their therapeutic efficiency when delivered locally while reducing leakage to the circulation and potential adverse effects. This effective strategy to engineer therapeutic proteins with a built-in delivery system has demonstrated to be effective in multiple models of tissue repair and regeneration and may also be applicable to those beyond regenerative medicine applications where local delivery is necessary to reach therapeutic efficacy at a low dose and limit off-target effects.

## Methods

### AREG fragment production

AREG fragments were cloned into the expression vector pGEX6P-1 (GE Healthcare) for expression in *E. coli* BL21 (DE3). Bacteria were cultured overnight in 10 ml lysogeny broth (LB) medium with 100 μg/ml of ampicillin. Then the culture was diluted 1:100 in 250 ml of LB medium with 100 μg/ml of ampicillin and cultured at 37 °C for 3 h. Protein production was induced with 1 mM of isopropyl β-D-1-thiogalactopyranoside (IPTG) overnight at 25 °C. The culture was then centrifuged at 4000 g for 10 min and pellets were resuspended in cold PBS with 1 tablet of protease inhibitor cocktail (Roche), 50 mg of lysozyme (Roche). The solution was sonicated for 20 s with maximum amplitude for 3-4 cycles. Benzonase (500 U, Millipore), 1 mM MgCl_2_, and 1% Triton X-100 were added, and the solution was incubated on a rotor for 30 min at 4 °C. Lysate was centrifuged at 12,000 g for 10 min and the supernatant filtered through a 0.22 μm filter. Proteins were first purified using a GSTrap HP 5 ml (GE Healthcare) affinity columns. Chaperone proteins were removed by using an ATP buffer (50 mM Tris-HCl, 150 mM NaCl, 10 mM MgSO_4_, 2 mM ATP, pH 7.4). A Triton X-114 buffer (PBS with 0.1% Triton X-114) was used to remove lipopolysaccharides. The final protein solution was dialysed against PBS and filtered through a 0.22 μm filter. The fragments were verified as >99% pure by SDS-PAGE and stored at −80 °C.

### Binding of protein candidates to ECM proteins and binding affinity measurements

ELISA plates (96-Well Medium Binding, Greiner bio-one) were coated for 1 h at 37 °C with 100 nM solutions of PlGF-2, AREG, NRTN, CXCL-9, CXCL-12 (SDF-1β), CXCL-17, VEGF-A, or BSA (Sigma, A8806). All recombinant proteins were carrier-free and purchased from Peprotech except CXCL-17 (R&D Systems). Wells were washed with washing buffer (phosphate-buffered saline with 0.05% Tween-20, PBS-T) and blocked with 1% BSA (Sigma, A8806) in PBS-T for 1 h at room temperature. Then, wells were incubated with 100 nM of human plasma fibronectin (Sigma), human vitronectin (Peprotech), human tenascin C (R&D Systems), or human fibrinogen (Enzyme Research Laboratories) in PBS-T with 0.1% BSA for 1 h at 37 °C. Wells were washed 3 times with washing buffer and incubated with for 1 h at room temperature with 0.1 μg/ml of HRP-conjugated antibody against fibronectin (Abcam, clone 5G7), vitronectin (Abcam, clone EP873Y), tenascin C (Abcam, clone EPR4219), or fibrinogen (Abcam, clone EPR3083). Wells were then washed three times with PBS-T and detection was done with tetramethylbenzidine (TMB) substrate and absorbance was measured at 450 nm. Results were expressed as fold change over BSA control. For the binding affinity measurements, ELISA plates were coated with PlGF-2, AREG, or NRTN at 100 nM for 1 h at 37 °C and further blocked with 1% BSA in PBS-T for 1 h at room temperature. Then, wells were incubated with ECM proteins at increasing concentration (50 μl in PBS-T containing 0.1% BSA). After washing the wells three times with PBS-T, binding was detected using the HRP-conjugated antibodies against ECM proteins. Dissociation constants (*K*_D_) were calculated using a one site specific binding model. A_450_ nm = B_max_*[ECM proteins]/(*K*_D_ + [ECM proteins]).

### Binding of AREG motif fragments to ECM proteins

ELISA plates (Medium Binding, Greiner bio-one) were coated for 1 h at 37 °C with 100 nM ECM proteins (including human collagen I (Chemicon, CC050) or II (Chemicon, CC052)). Wells were washed with washing buffer and blocked with 1% BSA in PBS-T for 1 h at room temperature. Then, wells were incubated with 100 nM of AREG motif fragment fused to GST in PBS-T with 0.1% BSA for 1 h at 37 °C. Wells were washed 3 times with washing buffer and incubated for 1 h at room temperature with HRP-conjugated antibody against GST (0.1 μg/ml in PBS-T with 0.1% BSA; Cytiva). Bound fragments were detected via TMB substrate and absorbance measurement at 450 nm.

### Binding affinity of AREG motif fragments to heparan sulfate

Heparin-binding plates (Corning, #354676) were coated with 25 μg/ml of heparan sulfate (Sigma-Aldrich, H7640) overnight at room temperature and blocked with a PBS solution containing 0.2% gelatin and 0.5% BSA for 1 h at room temperature. Wells were washed 3 times with washing buffer (100 mM NaCl, 50 mM NaAc, 0.2% Tween-20, pH 7.2) and GST-fused AREG fragments (100 nM in PBS with 0.5% BSA) were added. After 1 h incubation at room temperature, wells were washed three times with washing buffer and bound GST-fused AERG fragments were detected with the HRP-conjugated antibody against GST (0.1 μg/ml in PBS-T with 0.1% BSA). Wells were washed 3 times with PBS-T and detection was performed with TMB substrate and absorbance measured at 450 nm.

### Production of PDGF-BB and IL-1Ra variants

AREG_26-38_/PDGF-BB and AREG_26-38_/IL-1Ra were designed to have a 6x histidine tag. The histidine tag was inserted at the C-terminus for PDGF-BB and at the N-terminus of IL-1Ra. The AREG_26-38_ sequence was inserted at the N-terminus of both proteins. Recombinant proteins were produced in *E. coli* BL21 (DE3) via pET-22b (Novagen) as previously described^16^. Bacteria were cultured overnight in 10 ml LB medium with 100 μg/ml of ampicillin. Then the culture was diluted 1:100 in 1 L of LB medium with 100 μg/ml of ampicillin and incubated at 37 °C for 3 h. Protein production was induced with 1 mM of IPTG overnight at 25 °C. Then, the culture was centrifuged at 4000 *g* for 10 min. AREG_26-38_/PDGF-BB and PDGF-BB/PlGF_123-141_ were extracted from inclusion bodies using a solubilisation buffer (50 mM Tris, 6 M GuHCl, 10 mM DTT, pH 8.5). Extracted proteins were added in a refolding buffer (50 mM Tris, 1 mM GSH, 0.1 mM GSSG, pH 8.2) at 4 °C, over 4 days, for a final protein solution to buffer ratio of 1:100. Refolded proteins were then purified by affinity chromatography using a chelating SFF(Ni) column with an extensive Triton X-114 wash (0.1% v/v) to remove lipopolysaccharides. The fractions containing dimers were pooled together. Following protein production and bacterial lysis, the soluble fraction of AREG_26-38_/L-1Ra was purified by affinity chromatography using a chelating SFF(Ni) column with an extensive Triton X-114 wash (0.1% v/v) to remove lipopolysaccharides. AREG_26-38_/PDGF-BB was stored in 4 mM HCl and AREG_26-38_/IL-1Ra was stored in PBS with 5 mM EDTA. IL-1Ra and PDGF-BB were purchased from Peprotech.

### Binding affinity of AREG_26-38_-fused proteins to ECM proteins

ELISA plates (Medium binding, Greiner Bio-one) were coated with 100 nM of ECM proteins in 50 μl of PBS for 1 h at 37 °C. Then, wells were washed with PBS-T (PBS with 0.05% Tween-20) and blocked with 300 μl PBS-T containing 1% BSA for 1 h at room temperature. ECM and control wells (no ECM and blocked with BSA) were further incubated 1 h at room temperature with solutions of PDGF-BB variants or IL-1Ra variants at increasing concentrations (50 μl in PBS-T containing 0.1% BSA; PROKEEP tubes, Watson bio lab). Then, wells were washed three times with PBS-T and bound PDGF-BB and IL-1Ra variants were detected using biotinylated antibodies in PBS-T containing 0.1% BSA. Detection antibodies were from PDGF-BB DuoSet ELISA (R&D Systems, DY8464) for PDGF-BB and IL-1Ra/IL-1F3 DuoSet ELISA (R&D Systems, DY480) for IL-1Ra. To calculate the dissociation constants (*K*_*D*_) specific-binding values were fitted with a one site specific binding model using A_450_ nm = B_max_*[PDGF-BB or IL-1Ra variants]/(*K*_*D*_ + [PDGF-BB or IL-1Ra variants]).

### Release of AREG_26-38_-fused proteins from ECM-mimetic hydrogel

ECM-mimetic hydrogels (50 μl) were generated as previously described^16^. Briefly, the matrices were generated from HEPES solution (20 mM, 150 nM NaCl, pH 7.4) containing 8 mg/ml human fibrinogen, 1 mg/ml fibronectin, 500 μg/ml human vitronectin, 50 ug/ml tenascin C, 50 μg/ml heparan sulfate and 500 ng/ml of PDGF-BB or IL-1Ra variants. Matrices were polymerised in a 96-well plate (Corning) at 37 °C for 2 h with 10 U/ml bovine thrombin (Sigma) and 5 mM CaCl_2_. Then, matrices were transferred to Ultra Low Cluster 24-well plate (Corning) containing 500 μl of release buffer (20 mM Tris-HCl, 150 mM NaCl, 0.1% BSA, pH 7.4). Control wells that served as 100% released control contained only PDGF-BB and IL-1Ra variants in 500 μl of buffer. Every day, buffers were removed from wells and kept at -20 °C. Wells were replenished with fresh release buffer. For the 100% release control well, 20 μl of buffer was taken out every day and stored at −20 °C. After 7 days, the cumulative release of PDGF-BB and IL-1Ra variants was quantified by ELISA using the 100% released control as reference (PDGF-BB DuoSet, IL-1Ra/IL-1F3 DuoSet; R&D Systems). For release assays with plasmin, the same method was used but the release buffer contained 100 μU/ml of plasmin (Roche).

### Fibroblast proliferation assay

Tails of C57BL/6J mice were cut off from the base. An incision was made along the midline, throughout the length of the tail from the base to the tail tip, and the skin was peeled off from the bone. Tails were rinsed in PBS, cut into 1–2 cm^2^ pieces and incubated with 2 mg/ml ice-cold dispase II (Sigma) at 4 °C for 10–12 h. Then, skin pieces were washed, and the epidermis along with hair follicles was peeled off. Dermal pieces were minced and digested in with 300 U/ml collagenase II (Sigma) for 30 min at 37 °C. The supernatant was collected and filtered with a 100 μm filter and inactivated with EDTA (5 mM). Cells were centrifuged at 500 *g* for 10 min and plated on T75 flasks in fibroblast media (DMEM with 2 mM GlutaMAX, 1 mM sodium pyruvate, 10% heat-inactivated FBS, 100 units/ml penicillin and 100 mg/ml streptomycin). Cells were used within the first 3–4 passages for experiments. Fibroblasts were starved for 24 h in low-serum α-MEM (100 mg/ml penicillin/streptomycin, 2 mM GlutaMax, 2% FBS). Then, cells were seeded in a 96-well plate (2000 cells/well) with low-serum α-MEM containing PDGF-BB variants for 72 h. Percentage increase in cell number was calculated over basal proliferation (low-serum α-MEM only) using CyQuant Cell Proliferation Assay (Thermo Fisher Scientific).

### Release of IL-6 by macrophages

Bone marrow from C57BL/6J mice (8-week-old) was flushed out with Dulbecco’s Modified Eagle Medium/Nutrient Mixture (DMEM/F12 medium, Gibco) using a 27-gauge needle and a syringe. Cells were filtered through a 70 μm nylon strainer, centrifuged at 500 g for 10 min at 4 °C, and resuspended in DMEM/F12 medium containing 10% heat-inactivated FBS, 100 mg/ml penicillin/streptomycin, and 20 ng/ml murine M-CSF (Peprotech). Cells were plated in 150 mm diameter petri dishes at a density of 5 × 10^7^ and cultured for 7 d at 37 °C and 5% CO_2_. Medium was replaced every 3 days. After 7 days, macrophages were detached using TrypLE (Gibco) containing 3 mM EDTA and seeded in 12-well plates at a density of 2 × 10^5^ cells per well in DMEM/F12 with 10% heat-inactivated FBS and 1x penicillin/streptomycin (Thermo Fisher Scientific). Macrophages were co-stimulated with IL-1β (1 ng/ml) and IL-1Ra variants at increasing concentrations (0 to 1 μg/ml of IL-1Ra or equimolar concentration of AREG_26-38_/IL-1Ra). After 24 h, the concentration of IL-6 released in the media was measured by ELISA (IL-6 DuoSet ELISA kit, R&D Systems).

### Cleavage of GST-AREG_26-38_ by plasmin

GST or GST-AREG_26-38_ (50 μg) were incubated with plasmin (Roche) at increasing concentration in PBS for 1 h (total volume of 250 μl). The reaction was stopped by boiling the samples and cleavage was analysed by SDS-PAGE.

### Retention of AREG_26-38_-fused proteins in injured tissues

C57BL/6J mice (10-week-old) were used and mice were euthanised directly after (100% control) or at day 1, 3, 5, and 7 post-injection. For retention experiments after skin wounds, full-thickness punch-biopsy wounds (5 mm in diameter) were created. Then, 1 μg of wild-type IL-1Ra or AREG_26-38_/IL-1Ra was injected intradermally in four sites around the wound area (10 μl per injection). Full-thickness skin tissue around the wound was harvested with an 8 mm biopsy punch. For retention experiments in quadricep muscle defects, 4 μg PDGF-BB or an equimolar amount of AREG_26-38_/PDGF-BB was delivered in the quadriceps defect. Then, the entire quadriceps containing the fibrin matrix was harvested. In addition, one kidney, the left lateral liver lobe, the left lung lobe, and the spleen were collected at day 1, 3, 5, and 7 post-delivery. All harvested tissues were transferred into 500 μl of T-PER Tissue Protein Extraction Reagent (Thermo Fisher Scientific) containing a protease inhibitor cocktail (1 tablet for 50 ml, Roche) and minced. Samples were incubated for 1 h at room temperature under agitation, centrifugated at 5000 g for 5 min and supernatants were stored at −80 °C. Concentrations of proteins were determined by ELISA using an anti-histidine tag capture antibody (Abcam, ab18184) and a detection antibody from PDGF-BB or IL-1Ra/IL-1F3 DuoSet ELISA kit (R&D Systems). The day 0 samples were used to make a standard curve and to determine the 100% value. Protein concentrations in kidney, liver, lung, and spleen were calculated based on standard curves generated with the recombinant proteins.

### Animal ethics

Animal experiments were approved by the Monash Animal Research Platform ethics committee and by the Animal Research Committee of the Research Institute for Microbial Diseases of Osaka University (approval numbers 15216, 17075, 17731).

### Retention of AREG_26-38_-fused proteins in tissues

For intradermal retention, Male C57BL/6J mice (8-week-old) were anesthetised with isoflurane, shaved on the back, and 1 μg of 6 x histidine-tagged wild-type proteins (PDGF-BB, Elabscience; IL-1Ra, Sapphire Biosciences) or AREG_26-38_-fused proteins were injected intradermally. Injection sites were marked, and mice were euthanised directly after (100% control) or at day 1, 3, 5, and 7 post-injection. The area of the injection site was collected with a 5 mm biopsy punch (full-thickness skin tissue). For intramuscular injection, 1 μg of wild-type or AREG_26-38_-fused proteins were injected in the tibialis anterior muscle and then the entire muscle was collected at the different time points. Harvested tissues were transferred into 500 μl of T-PER Tissue Protein Extraction Reagent (Thermo Fisher Scientific) containing a protease inhibitor cocktail (1 tablet for 50 ml, Roche) and minced. Samples were incubated for 1 h at room temperature under agitation, centrifuged at 5000 *g* for 5 min and supernatants were stored at -80 °C. Concentration of proteins were determined by ELISA using an anti-histidine tag capture antibody (Abcam, ab18184) and a detection antibody from PDGF-BB or IL-1Ra/IL-1F3 DuoSet ELISA kit (R&D Systems). The day 0 samples were used to make a standard curve and to determine the 100% value. Four mice per group per time point were used in all retention experiments.

### Tumour growth model

E.G7-OVA thymoma cells were obtained from American Type Culture Collection (ATCC) and grown as previously described. Tumour cells (1 × 10^6^) were injected subcutaneously in the back of male C57BL/6J mice (8-week-old) in 30 μl of saline, following anaesthesia with isoflurane. Four and six days later, mice were injected with PDGF-BB variants (10 μg per injection in 30 μl of saline) or saline subcutaneously 1 cm away from the tumour injection sites. Tumours were measured every day and volumes were calculated as ellipsoids based on three orthogonal measures as described previously^67^. Animals were monitored for 14 days. Animals having a tumour volume > 1000 mm^3^ before 14 days were humanely culled. Six mice per group were used.

### Measurement of AREG_26-38_-fused protein serum concentrations

PDGF-BB variants were injected subcutaneously (10 μg per injection in 30 μl of saline) in C57BL/6J mice (8-week-old). Blood (100 μl) was collected at different time points by tail bleeding in anticoagulant coated tubes (Eppendorf). Directly after collection samples were centrifuged for 15 min at 1000 *g*. Plasma concentrations of PDGF-BB variants were detected by ELISA (Mouse/Rat PDGF-BB Quantikine ELISA Kit, R&D Systems).

### Wound healing model

BKS.Cg-*Dock7*^*m*^ +/+ *Lepr*^*db*^/J (*Lepr*^*db/db*^) mice were obtained from the Jackson Laboratory. Male C57BL/6J mice (12 to 14-week-old) were anesthetised with isoflurane and shaved on the back. For analgesia, 0.1 mg/kg buprenorphine was administered subcutaneously. Two full-thickness punch-biopsy wounds (5 mm in diameter) were created as described previously^36^. The following day, wounds were treated topically with 10 μl saline (PBS) or PDGF-BB variants (0.5 μg PDGF-BB or equimolar amount of AREG_26-38_/PDGF-BB). Wounds were covered with a non-adherent dressing (Adaptic, Johnson & Johnson), spot bandages and secured with adhesive film dressing (Hydrofilm, Hartmann). Animals were humanely culled by CO_2_ asphyxiation. Wounds were harvested for biochemical or histological analysis with an 8 mm tissue biopsy punch. Samples were fixed in 10% neutral buffered formalin for 24 h at room temperature. Wounds were cut at the edge of the closure, embedded in paraffin, and sectioned at 4 μm onto slides until the centre of the wound was passed. Re-epithelialisation was then measured by histomorphometric analysis. Slides were stained with haematoxylin and eosin and the centre of the wound was determined by measuring the distance between the panniculus carnosus muscle gap using Aperio ImageScope Viewer (Germany). Closure was calculated as the ratio between the epidermis closure over the length of the panniculus carnosus gap. Eight to ten wounds per group per time point were used.

### Immunostaining of wound sections

Immunostaining was performed on paraffin sections with a sodium citrate (10 mM sodium citrate, 0.05% Tween-20, pH 6) antigen retrieval step. Slides were washed with 0.05% Tween-20 (PBS-T) for 5 min followed by a permeabilisation step in 0.01 M PBS containing 0.2% Triton X-100 for 4 min at room temperature. Sections were washed with PBS-T and blocked with 1% BSA with 10% normal goat serum (NGS) for 2 h at room temperature. Sections were incubated with rabbit anti-CD31 (0.8 μg/ml Abcam, ab124432) and mouse anti-desmin (1 μg/ml, Abcam, ab8470) in 0.1% BSA with 1% NGS in 0.01 M PBS (antibody buffer) overnight at 4 °C. Sections were washed with PBS-T and incubated with biotinylated goat anti-rabbit IgG (1μg/ml, Thermo Fisher Scientific, B2770) in antibody buffer for 2 h at room temperature followed by streptavidin Alexa Fluor 594 (2 μg/ml, Life Technologies, S11227) and goat anti-Mouse IgG Alexa Fluor 488 (2 μg/ml, abcam, ab150117) in antibody buffer for 2 h at room temperature. Sections were washed with PBS-T, nucleus stained with 4’,-6-Diamidino-2-phenylindole dihydrochloride (DAPI) (1 μg/ml, Sigma Aldrich D9542-10MG) for 15 min at room temperature, and mounted with Fluoroshield (Sigma Aldrich, F6182). Measurement was performed by taking images of three different regions from the middle of the wound area covering the dermis and epidermis (Zeiss Z1 Inverted Fluorescence Microscope) at a 20x magnification. The area positive for CD31 was measured using ImageJ software (National Institutes of Health, USA).

### Detection of PDGF receptors by western blot

Primary human skeletal muscle satellite cells (Lifeline Cell Technology) and human umbilical vein endothelial cells (Sigma-Aldrich) were grown to 70% confluency in StemLife Sk Complete Medium (Lifeline Cell Technology) and endothelial cell growth medium (Lonza), respectively. Cells were rinsed with PBS and lysed using RIPA buffer (150 mM NaCl, 1% nonidet P-40, 0.5% sodium deoxycholate, 0.1% sodium dodecyl sulfate, 50 mM Tris pH 8.0) containing protease and phosphatase inhibitor cocktails (Roche). Lysates were resolved by SDS-PAGE using a 4-20% gradient gel (Bio-Rad), then electrotransferred onto a PVDF membrane. Membranes were blocked with 5% skim milk powder in PBS-T. Wash steps were performed with PBST. Membranes were probed with rabbit anti-PDGFRα (clone (D1E1E, Cell Signalling Technology, 1:1000 dilution), rabbit anti-PDGFRβ (clone 28E1, Cell Signalling Technology, 0.025 μg/ml) and rabbit anti-Actin (ab200658, Abcam, 0.025 μg/ml). Primary antibodies were diluted in 3% BSA in PBS-T. After washing, membranes were incubated with HRP-conjugated goat anti-rabbit IgG (Thermo Fisher Scientific, 0.25 μg/ml). Secondary antibody was diluted in 5% skim milk powder in PBS-T. Protein bands were visualised by chemiluminescence after incubation with chemiluminescent peroxidase substrate (Sigma-Aldrich) and imaging using a G:Box gel dock (Syngene). All blots derive from the same experiment and were processed in parallel.

### Human muscle satellite cell proliferation assay

Primary human skeletal muscle satellite cells (Lifeline Cell Technology) were plated at a density of 1000 cells per well containing 100 μl of StemLife Sk Complete Medium (Lifeline Cell Technology) in a 96-well plate and cells were incubated for 24 h at 37 °C, 5% CO_2_. Then 100 μl StemLife Basal Medium (Lifeline Cell Technology) containing PBS (negative control) or recombinant human PDGF-BB (PeproTech) was added to each well to achieve a total volume of 200 μl per well. PDGF-BB treatment was performed with 50 pM, 200 pM, 1 nM or 2 nM final concentrations per well. Six replicate wells were used per treatment condition. Cells were incubated for a further 48 h, after which all media was removed by aspiration and the 96-well plate frozen at -80 °C to enable cell lysis. After freezing, plates were thawed at room temperature and cell density was measured using the CyQuant Cell Proliferation Assay kit (Thermo Fisher Scientific) according to manufacturer’s instructions. Briefly, 200 μl of cell lysis buffer containing CyQuant GR dye (1:400) was added to each well and incubated at room temperature for 5 min, then fluorescence was analysed by fluorescence microplate reader (480 nm excitation, 520 nm emission) (BioTek Synergy H1, Agilent Technologies Inc.). Proliferation increase for each PDGF-BB treated condition was calculated as percentage increase in average fluorescence value versus the PBS treated condition.

### Volumetric muscle loss model

Male C57BL/6J mice (10-week-old) were anesthetised with isoflurane and shaved on the hind left leg. For analgesia, 0.1 mg/kg buprenorphine was administered subcutaneously. A unilateral incision measuring approximately 1 cm was made exposing the underlying fascia. The left hind limb was extended and exteriorised via the incision site by retracting the surrounding tissue. The muscle was excised by 7 mm (distal form a patella) x 3 mm (from vastus intermedius). Directly after, the injury site was covered with fibrin hydrogel containing 4 μg PDGF-BB or an equimolar amount of AREG_26-38_/PDGF-BB that polymerised in the defect (80 μl, 8 mg/ml human fibrinogen (FIB3, Enzyme Research Laboratories), 4 U/ml bovine thrombin (T4648, Sigma), 5 mM CaCl_2_, 17 μg/ml of aprotinin (ab146286, Abcam)). Then, the soft tissue was closed with staples.

Animals were humanely culled by cervical dislocation. Mouse legs without the skin were harvested after three weeks and fixed in 10% formalin for 24 h at room temperature. Next day, legs were soaked in 3% nitric acid for 10 h for decalcification before the routine tissue process. Muscle tissues were embedded in paraffin and sectioned into 5-μm-thick sagittal sections. Muscle tissue sections were stained with Masson’s Trichrome and analysed using Aperio ImageScope Viewer (Germany). Multiple sectioning depths were used to find the centre of the muscle indicated by the position of the femur and patella. The area of muscle tissue at the centre was measured based on the initial defect area (7 mm distal to the patella and 1 mm above the femur bone). The same measurement was done on the uninjured control left leg of the mouse. Muscle tissue restoration was calculated as a percentage of the uninjured control right leg. Seven mice per group were used.

### Myocardial infarction model

Male C57BL/6J mice (10-week-old) where anesthetised with isoflurane and attached to an artificial respirator by endotracheal cannulation. For analgesia, 0.05 mg/kg buprenorphine was administered subcutaneously. The artificial respirator maintained isofluorane concentration at 2% vol/vol with 100% O_2_ and operated at a stroke volume of 200 μl at 120 strokes per min. An oblique 8 mm incision was made 2 mm away from the left sternal border toward the left armpit to reveal pectoral muscles. An incision was made through the muscle of the 4^th^ intercostal space. The left coronary artery (LCA) was visualised as a pulsating bright red spike. Using a micro-needle holder, a 6 mm tapered needle with 8–0 polyethylene suture was passed through the myocardium underneath the LCA and the ligature was tied with three knots. Occlusion was confirmed by the change of colour of the anterior wall of the left ventricle. IL-1Ra variants were resuspended in saline at a concentration of 0.2 μg/μl. For mice receiving intramyocardial injections, a 25 μl Hamilton gas tight syringe with a 30 G needle was used to inject a total of 20 μl (5 μl in 4 sites for a total of 4 μg) at the border of the infarct. Mouse thoracic cavity, and skin incision were closed and sutured. For mice receiving systemic injections, 40 μg of IL-1Ra was delivered intraperitoneally. Immediately after surgical procedure, the mice were transferred to a heated pad in a supine position and connected to an artificial respirator pumping air without isoflurane to allow the mice to regain consciousness. Once mice became conscious, the cannula was removed, and the mice were placed on a heating pad for 24 h. Seven mice per group were used.

### Echocardiography

Cardiac ultrasound analysis was performed on Vevo 2100 system, as previously described^68^. On day 1 (baseline), day 7 and day 28, the animals were scanned under light anaesthesia with isoflurane (1.5 vol%). All hair in the thoracic region was removed using a depilatory agent and ultrasound gel was applied to the thoracic region to improve sound wave transmission. Two-dimensional imaging (B-mode) was performed to obtain a parasternal short axis view and a long axis view. For long-axis B-mode measurements the endocardium was traced from the mitral valve, excluding the papillary muscle. Echocardiographic measurements of the left ventricle included ejection fraction, fractional shortening, end-diastolic dimension, end-systolic dimension, and anterior wall thickness were calculated by the Vevo 2100 software using standard computational methods.

### Histopathological and immunofluorescence analyses for the myocardial infarction model

Animals were euthanised by cervical dislocation. Mouse hearts were excised, briefly washed in PBS, and fixed in 10% formalin at room temperature. Formalin-fixed tissues were embedded in paraffin, cut into 4 µm tissue sections and placed onto slides. Paraffin tissue sections were processed for Masson’s Trichrome staining using standard histological procedures. Infarct size was calculated as the mean of seven sections calculated as a percentage of the infarct area to total left ventricular circumference, using NIH ImageJ software. Three days post-treatment myocardial apoptosis was examined using the In Situ Cell Death Detection Kit, TMR red (Roche, #12156792910). Sections were permeabilised in 0.2% Triton X-100 in PBS for 8 min. Sections were washed with PBS and 50 µl of the terminal deoxynucleotidyl transferase dUTP nick end labelling (TUNEL) reaction mixture was added on each heart section for 1 h at 37 °C. Slides were washed with HBSS (Hank’s Buffered Salt Solution) and incubated with wheat germ agglutinin (WGA) conjugated with FITC (Sigma-Aldrich, # L4895, 20 µg/ml) for 2 h at room temperature followed by DAPI staining. Two slides from each block were evaluated for percentage of apoptotic cells and three fields on each slide were examined at the border areas. The cardiomyocytes were considered apoptotic if the TUNEL staining was in the middle of a cell with its borders stained by WGA. Four mice per group were used.

### Apoptosis of human cardiomyocytes

Human iCell cardiomyocytes (#01434, Fujifilm Cellular Dynamics) were maintained according to the manufacturer’s instructions. Cardiomyocytes were seeded in 8-well chamber slides (#154941, Thermo Fisher Scientific) coated with plasma fibronectin (Sigma-Aldrich) at the density of 150,000 cells/cm^2^ at 37 °C, in 5% CO_2_ for 24 h. The Plating Medium was changed with Maintenance Medium (Fujifilm Cellular Dynamics) and replaced every 48 h. On day 5 post-seeding, human IL-1β (PeproTech) was added and cells were incubated for 72 h. Cell apoptosis was assessed using In Situ Cell Death Detection Kit (Sigma-Aldrich) or Image-iT LIVE Caspase Apoptosis Detection Kits (#I35101, Thermo Fisher Scientific). Images were captured at a 10x magnification (Leica, AF6000LX) and quantification of the number of apoptotic cells was performed using ImageJ software (National Institutes of Health, USA).

### Statistical analysis

All data are presented as means ± SEM, otherwise indicated. Statistical analyses were performed using GraphPad Prism 9 statistical software (GraphPad, USA). Significant differences were calculated with Student’s *t* test, by analysis of variance (ANOVA), followed by Bonferroni *post hoc* test when performing multiple comparisons between groups, or by one-sample *t* test. *P* < 0.05 was considered as a statistically significant difference. The *P* values are indicated and the symbol *** indicates *P* values equal or less than 0.001; n.s. indicates not significant.

## Supplementary information

SUPPLEMENTAL MATERIAL

Supplementary Movie 1

Supplementary Movie 2

Supplementary Movie 3

Supplementary Movie 4

## Data Availability

The data that support the findings of this study are available from the corresponding author upon reasonable request.
