# Supplementary material for: A superior extracellular matrix binding motif to enhance the regenerative activity and safety of therapeutic proteins
Source: NPJ Regen Med. 2023 May 22;8:25. doi: 10.1038/s41536-023-00297-0 (PMC10202959; doi:10.1038/s41536-023-00297-0)

Supplementary information

A superior extracellular matrix binding motif to enhance the regenerative activity and safety of therapeutic proteins

Yasmin K. Alshoubaki[1†], Yen-Zhen Lu[1†], Julien M.D. Legrand[1], Rezvan Karami[1], Mathilde Fossat[1], Ekaterina Salimova[2], Ziad Julier[1], Mikaël M. Martino[1,3,4*].

Affiliations:

[1]European Molecular Biology Laboratory Australia, Australian Regenerative Medicine Institute, Monash University, Melbourne, VIC 3800, Australia

[2]Monash Biomedical Imaging, Monash University, Clayton, VIC 3800, Australia

[3]Victorian Heart Institute, Monash University, Clayton, VIC 3800, Australia

[4]Laboratory of Host Defense, World Premier Institute Immunology Frontier Research Center, Osaka University, Osaka, Japan

The PDF file includes:

Supplementary Figures 1-10

Supplementary Tables 1-3

Supplementary Movie 1-4 legend

Unprocessed Western Blots

1	|Q12904|AIMP1 (312 aa)	51	|Q9UK53|ING1 (422 aa)	101	|Q9BRC7-2|PLCD4 (272 aa)	151	|Q96HF1|SFRP2 (295 aa)
2	|Q12904-2|AIMP1 (336 aa)	52	|Q9UK53-2|ING1 (279 aa)	102	**|P49763-3|PLGF (170 aa)**	152	|O75264|SIM24 (130 aa)
3	|Q66PJ3|AR6P4 (237 aa)	53	|Q9UK53-3|ING1 (210 aa)	103	**|P49763-4|PLGF (242 aa)**	153	|A0A1B0GVY4|SIM31 (71 aa)
4	|Q66PJ3-1|AR6P4 (421 aa)	54	|Q9UK53-4|ING1 (235 aa)	104	|A0A1B0GUJ8|PNM8C (204 aa)	154	|Q96A28|SLAF9 (289 aa)
5	|Q66PJ3-10|AR6P4 (215 aa)	55	|Q9UK53-5|ING1 (262 aa)	105	|A8MPX8-2|PP2D1 (368 aa)	155	|Q96A28-2|SLAF9 (198 aa)
6	|Q66PJ3-2|AR6P4 (413 aa)	56	|Q9H160|ING2 (280 aa)	106	|A8MPX8-3|PP2D1 (376 aa)	156	|Q9H4F8|SMOC1 (434 aa)
7	|Q66PJ3-3|AR6P4 (402 aa)	57	|Q9H160-2|ING2 (240 aa)	107	|Q13427-2|PPIG (357 aa)	157	|Q9H4F8-2|SMOC1 (435 aa)
8	|Q66PJ3-4|AR6P4 (399 aa)	58	|Q9UNL4|ING4 (249 aa)	108	|Q8WUA2|PPIL4 (492 aa)	158	|Q9H3U7|SMOC2 (446 aa)
9	|Q66PJ3-5|AR6P4 (181 aa)	59	|Q9UNL4-2|ING4 (248 aa)	109	|P35813-3|PPM1A (455 aa)	159	|Q9H3U7-2|SMOC2 (457 aa)
10	|Q66PJ3-7|AR6P4 (226 aa)	60	|Q9UNL4-3|ING4 (225 aa)	110	|Q8NAV1|PR38A (312 aa)	160	|Q9HD40-3|SPCS (500 aa)
11	|Q66PJ3-9|AR6P4 (218 aa)	61	|Q9UNL4-6|ING4 (248 aa)	111	|Q8NAV1-2|PR38A (125 aa)	161	|Q8N9Q2|SR1IP (155 aa)
12	**|P15514|AREG (252 aa)**	62	|Q9UNL4-7|ING4 (246 aa)	112	|P04554|PRM2 (102 aa)	162	|Q9UQ35-3|SRRM2 (311 aa)
13	|Q9NWB6|ARGL1 (273 aa)	63	|Q9UNL4-8|ING4 (179 aa)	113	|P04554-2|PRM2 (140 aa)	163	|Q01130|SRSF2 (221 aa)
14	|Q9NWB6-2|ARGL1 (273 aa)	64	|Q8WYH8|ING5 (240 aa)	114	|Q8WWY3|PRP31 (499 aa)	164	|Q13247|SRSF6 (344 aa)
15	|Q8TDN6|BRX1 (353 aa)	65	|Q8WYH8-2|ING5 (226 aa)	115	|Q8WWY3-4|PRP31 (491 aa)	165	|Q13247-3|SRSF6 (335 aa)
16	|D4N3P3|CAPSD_HCYV5 (219 aa)	66	|Q8IZA0-3|K319L (491 aa)	116	|Q9NZ81|PRR13 (148 aa)	166	|O75683|SURF6 (361 aa)
17	|Q9H6E4|CC134 (229 aa)	67	|Q9Y383|LC7L2 (392 aa)	117	|Q9NZ81-2|PRR13 (98 aa)	167	|Q7L8C5|SYT13 (426 aa)
18	|Q96MF4|CC140 (163 aa)	68	|Q9Y383-2|LC7L2 (391 aa)	118	|Q96B01|R51A1 (352 aa)	168	|Q86TJ2|TAD2B (420 aa)
19	|Q8TD26-2|CHD6 (373 aa)	69	|Q9Y383-3|LC7L2 (389 aa)	119	|Q96B01-2|R51A1 (335 aa)	169	|Q86TJ2-2|TAD2B (345 aa)
20	|Q5T280|CI114 (376 aa)	70	|O95232|LC7L3 (432 aa)	120	|Q96B01-3|R51A1 (302 aa)	170	|Q86TJ2-3|TAD2B (328 aa)
21	|Q86X95|CIR1 (450 aa)	71	|Q8N3X6-2|LCORL (213 aa)	121	|P42696|RBM34 (430 aa)	171	|B6A8C7-2|TARM1 (279 aa)
22	|P49711-2|CTCF (399 aa)	72	|Q6ZQX7|LIAT1 (453 aa)	122	|Q9Y388|RBMX2 (322 aa)	172	|Q92664|TF3A (365 aa)
23	|Q9NXE8|CWC25 (425 aa)	73	|Q6ZQX7-2|LIAT1 (423 aa)	123	|Q07020|RL18 (188 aa)	173	|Q92664-2|TF3A (340 aa)
24	**|Q07325|CXCL9 (125 aa)**	74	|Q6ZQX7-3|LIAT1 (433 aa)	124	|Q07020-2|RL18 (159 aa)	174	|Q86V40-2|TIKI1 (456 aa)
25	**|Q6UXB2|CXL17 (119 aa)**	75	|Q6ZQX7-4|LIAT1 (423 aa)	125	|P84098|RL19 (196 aa)	175	|Q8TBZ6|TM10A (339 aa)
26	|Q14093|CYLC2 (348 aa)	76	|Q9BRT6|LLPH (129 aa)	126	|P62750|RL23A (156 aa)	176	|Q9NW97|TMM51 (253 aa)
27	|Q8NBI3|DRAXI (349 aa)	77	|Q8N309-2|LRC43 (471 aa)	127	|P62945|RL41 (25 aa)	177	|Q5VYS8-5|TUT7 (412 aa)
28	|O00472-2|ELL2 (390 aa)	78	|Q9NQ29|LUC7L (371 aa)	128	|P46777|RL5 (297 aa)	178	|P26368|U2AF2 (475 aa)
29	|Q70Z53|F10C1 (315 aa)	79	|Q9NQ29-2|LUC7L (325 aa)	129	|Q9P015|RM15 (296 aa)	179	|P26368-2|U2AF2 (471 aa)
30	|Q70Z53-2|F10C1 (276 aa)	80	|Q9NQ29-3|LUC7L (354 aa)	130	|Q8IXM3|RM41 (137 aa)	180	|Q15696|U2AFM (482 aa)
31	|Q70Z53-3|F10C1 (315 aa)	81	|Q9NX58|LYAR (379 aa)	131	|Q8TA86|RP9 (221 aa)	181	**|P15692|VEGFA (232 aa)**
32	|Q70Z53-4|F10C1 (292 aa)	82	|A0JLT2|MED19 (244 aa)	132	|P62847|RS24 (133 aa)	182	**|P15692-13|VEGFA (395 aa)**
33	|Q70Z53-5|F10C1 (313 aa)	83	|Q9BU76|MMTA2 (263 aa)	133	|P62847-2|RS24 (130 aa)	183	**|P15692-14|VEGFA (412 aa)**
34	|Q8N9E0|F133A (248 aa)	84	|O60524-2|NEMF (276 aa)	134	|P62847-3|RS24 (132 aa)	184	**|P15692-16|VEGFA (389 aa)**
35	|Q5BKY9|F133B (247 aa)	85	|Q9NX24|NHP2 (153 aa)	135	|P62847-4|RS24 (289 aa)	185	**|P15692-2|VEGFA (215 aa)**
36	|Q5BKY9-2|F133B (237 aa)	86	|Q6ZUT1|NKAP1 (292 aa)	136	|Q6UXX9|RSPO2 (243 aa)	186	**|P15692-3|VEGFA (209 aa)**
37	|Q9UHL3|F153A (310 aa)	87	|Q6ZUT1-2|NKAP1 (293 aa)	137	|Q6UXX9-2|RSPO2 (176 aa)	187	**|P15692-6|VEGFA (171 aa)**
38	|P0C7A2|F153B (387 aa)	88	|Q6ZUT1-3|NKAP1 (264 aa)	138	|Q6UXX9-3|RSPO2 (179 aa)	188	|Q9UPY6-2|WASF3 (499 aa)
39	|Q96PV7-2|F193B (448 aa)	89	|Q5M9Q1|NKAPL (402 aa)	139	|Q9BXY4|RSPO3 (272 aa)	189	|Q9NP64|ZCC17 (241 aa)
40	|Q9NYL4|FKB11 (201 aa)	90	|Q8N5F7|NKAP (415 aa)	140	|Q9BXY4-2|RSPO3 (292 aa)	190	|Q9NP64-2|ZCC17 (217 aa)
41	|Q96HJ9-2|FMC1 (458 aa)	91	|P30419|NMT1 (496 aa)	141	|Q2I0M5|RSPO4 (234 aa)	191	|Q9NP64-3|ZCC17 (233 aa)
42	|Q8N0W7|FMR1N (255 aa)	92	|O60551|NMT2 (498 aa)	142	|Q2I0M5-2|RSPO4 (172 aa)		
43	|Q8TAE8|G45IP (222 aa)	93	**|Q99748|NRTN (197 aa)**	143	|Q96IZ7|RSRC1 (334 aa)		
44	|O60755|GALR3 (368 aa)	94	|Q9H1E3|NUCKS (243 aa)	144	|P82675|RT05 (430 aa)		
45	|Q99680|GPR22 (433 aa)	95	|Q9H1E3-2|NUCKS (203 aa)	145	|P82675-2|RT05 (251 aa)		
46	|Q9BXL5|HEMGN (484 aa)	96	|Q8N8D1|PDCD7 (485 aa)	146	|Q9BY42|RTF2 (306 aa)		
47	|O94992|HEXI1 (359 aa)	97	|Q86YI8|PHF13 (300 aa)	147	|P08621|RU17 (437 aa)		
48	|Q96MH2|HEXI2 (286 aa)	98	|Q9BUL5|PHF23 (403 aa)	148	|P08621-2|RU17 (428 aa)		
49	|P17096-3|HMGA1 (179 aa)	99	|Q9BUL5-3|PHF23 (336 aa)	149	|P08621-4|RU17 (341 aa)		
50	|P55010|IF5 (431 aa)	100	|Q9BUL5-4|PHF23 (399 aa)	150	**|P48061-3|SDF1 (119 aa)**		

Supplementary Table 1. List of proteins found using the search motif against the human protein database. The primary accession number, name, and the number of amino acids (aa) are indicated. Growth factors and cytokines are highlighted in red and bold.

```
PlGF123-138 RRRPKGR-GKRRREKQR    PlGF123-137 RRRPKGRGKRRREKQ-    PlGF128-138 ------RGKRRREKQR
W-E=42      :..  :. :: ::....    W-E=34      ...  :. ::.....     W-E=24            . ...:....
AREG26-42   RKKKGGKNGKNRRNRKK    CXCL-1276-80 KKEKIGKKKRQKKRK-   CXCL-1766-76 ------KTRHQRHHRK

PlGF123-138 -RRPKGRGKRRREKQR     PlGF124-137 -RRPKGRGKRRREKQ-    PlGF126-138 ----KGRGKRRREKQR
W-E=42       :: . : . :::. :     W-E=31       .. .   ::....:.    W-E=24          : :....... :
NRTN51-66   -RRLRQRRRLRRERVR     CXCL-974-87 -KKKQKNGKKHQKKK-    VEGF-A108-119 ----KDRARQEKKSVR
```

Supplementary Fig. 1. Local pairwise sequence alignments between PIGF$_{123\text{-}138}$ and the motif from protein candidates using the Smith-Waterman algorithm. Two dots indicate same amino acids and one dot indicates amino acid with similar proprieties. Waterman-Eggert score (N-E) is shown.

	Fibronectin	Vitronectin	Tenacin C	Fibrinogen
PlGF K_D (nM)	17.2 ± 1.8	13.2 ± 4.0	79.4 ± 2.5	14.1 ± 3.5
AREG K_D (nM)	8.9 ± 0.5	9.3 ± 1.2	11.0 ± 1.0	7.4 ± 1.5
NRTN K_D (nM)	79.4 ± 17.3	53.1 ± 12.8	55.7 ± 12.8	389.7 ± 42.7

Supplementary Table 2. Binding-affinity of PlGF, AREG and NRTN to ECM proteins.

Dissociation constants (K_D) in nM are shown. Data are means ± SEM. n = 3.

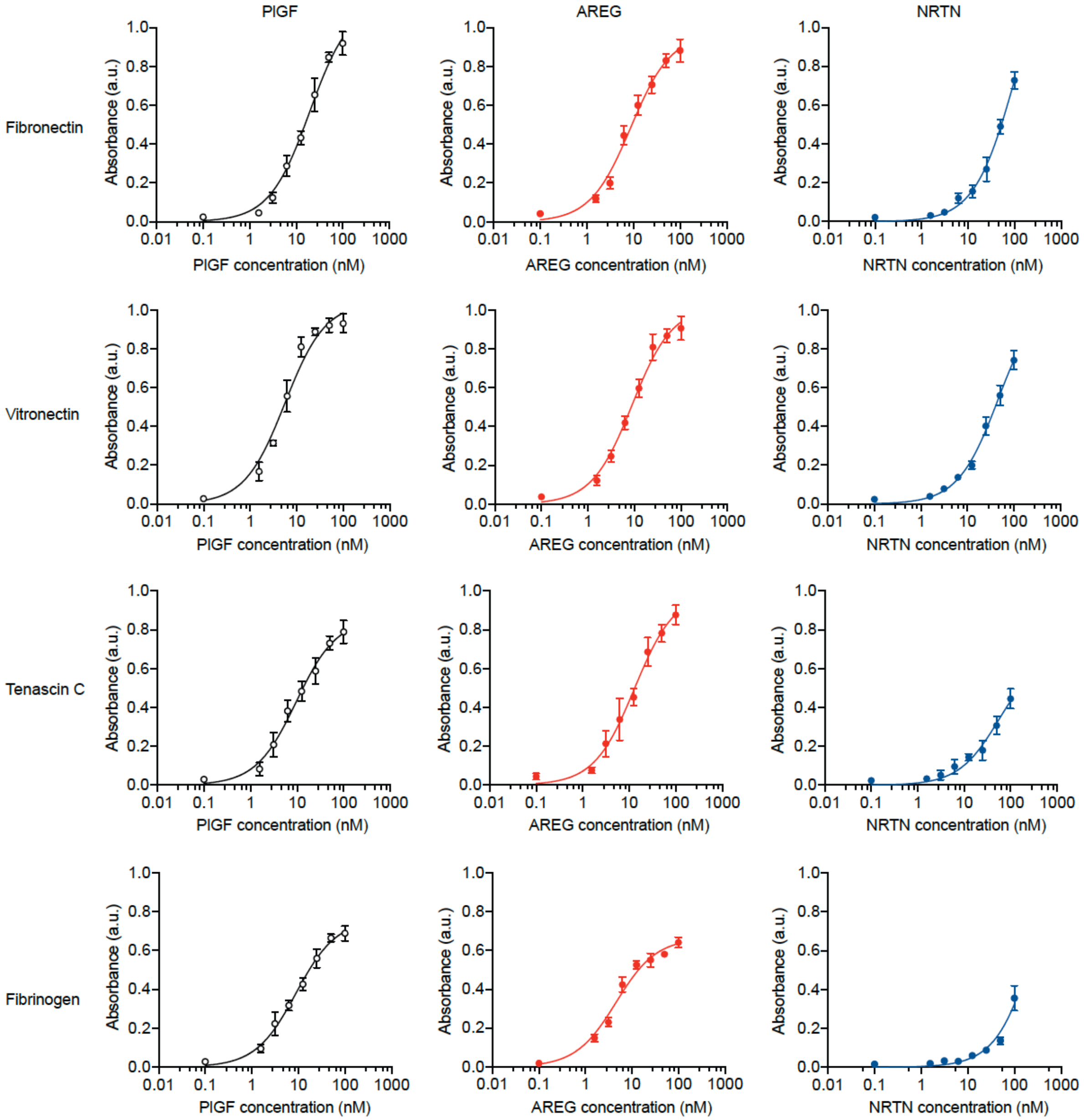

Supplementary Fig. 2. Binding curves of PlGF, AREG and NRTN to ECM proteins. ELISA plate wells were coated PlGF, AREG or NRTN and further incubated with ECM proteins at increasing concentrations. Graphs show signals given by antibodies detecting ECM proteins. The signals were fitted by non-linear regression to obtain the dissociation constant (K_D) using A450 nm = B_{max}*[protein]/(K_D + [protein]) where [protein] is the concentration of PlGF, AREG or NRTN. Representative binding curves are shown. Data are means ± SD.

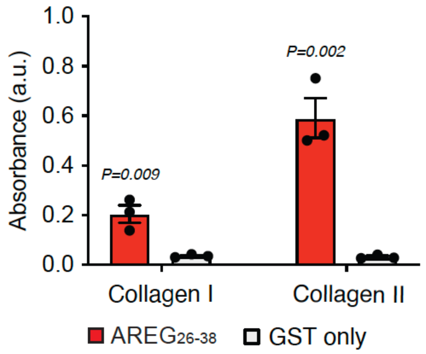

Supplementary Fig. 3. Binding of $AREG_{26\text{-}38}$ to collagen I and II. ELISA plates were coated with collagen I or II and incubated with GST-fused $AREG_{26\text{-}38}$ or GST only. Graphs show signals given when detecting GST. *n* = 3. Data are means ± SEM. Two-tailed Student's t-test. *P* values are indicated.

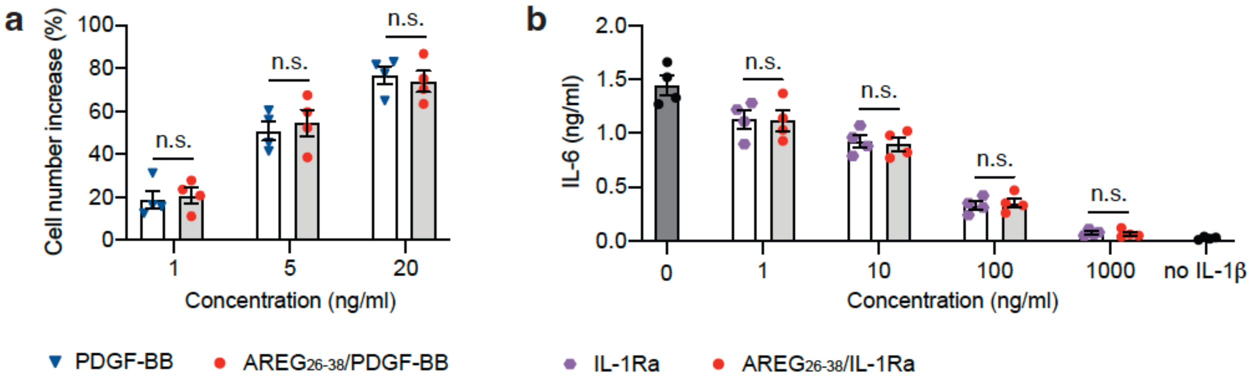

Supplementary Fig. 4. Fusion of AREG$_{26\text{-}38}$ to PDGF-BB and IL-1Ra does not impair activity. **a** Dermal fibroblasts were cultured in basal media (2% serum) and stimulated with PDGF-BB variants at increasing concentrations. The percentage increase in cell number was measured after 3 days. *n* = 4. **b** Bone marrow-derived macrophages were co-stimulated with IL-1β (1 ng/ml) and IL-1Ra variants at increasing concentrations (0 to 1 μg/ml). The negative control was no IL-1β treatment. The ability of IL-1Ra variants to inhibit IL-1β was assessed by measuring the release of IL-6 by macrophages 24 h after stimulation. *n* = 4. For both panels data are means ± SEM. Two-tailed Student's t-test. n.s. = non-significant.

	Fibronectin	Vitronectin	Tenacin C	Fibrinogen
PDGF-BB K_D (nM)	74.2 ± 7.1	29.7 ± 3.1	87.8 ± 4.7	209.7 ± 20.3
$AREG_{126\text{-}138}$/PDGF-BB K_D (nM)	5.9 ± 1.1	1.3 ± 0.3	19.3 ± 1.1	13.9 ± 7.4
IL-1Ra K_D (nM)	153.3 ± 9.7	96.5 ± 9.4	137.1 ± 8.8	640.1 ± 45.5
$AREG_{126\text{-}138}$/IL-1Ra K_D (nM)	16.5 ± 2.7	1.9 ± 1.6	18.6 ± 2.4	16.9 ± 2.3

Supplementary Table 3. Binding-affinity of $AREG_{26\text{-}38}$-fused therapeutic proteins to ECM proteins. Dissociation constants (K_D) in nM are shown. Data are means ± SEM. n = 3.

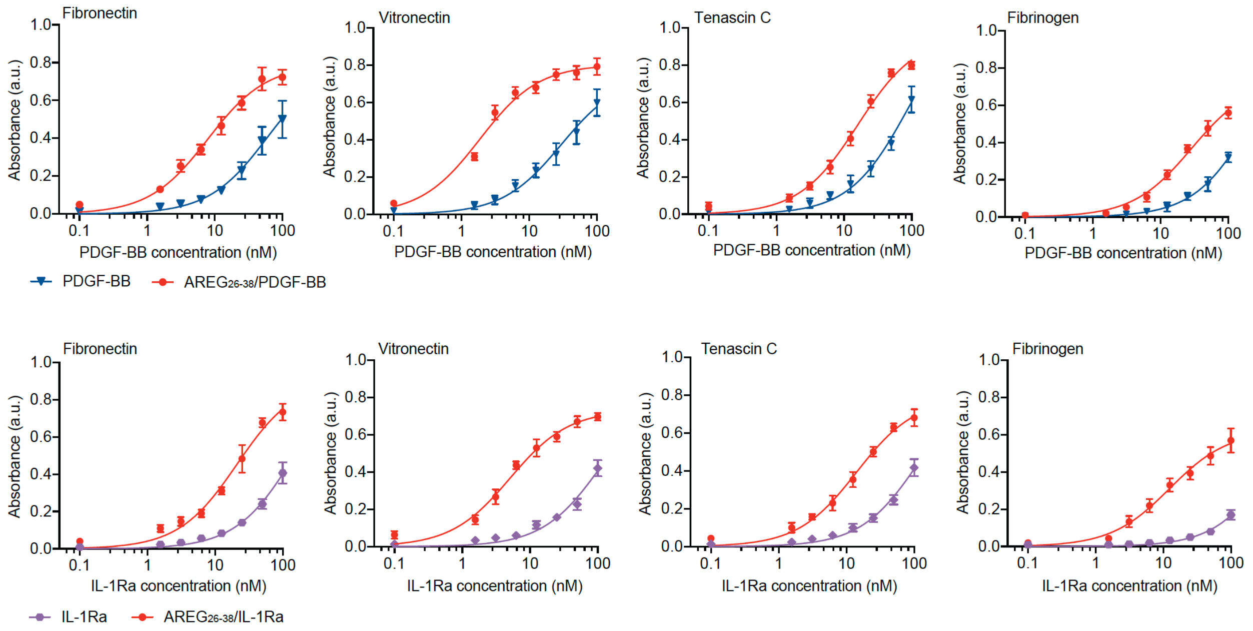

Supplementary Fig. 5. Binding curves of AREG$_{26\text{-}38}$-fused therapeutic proteins to ECM proteins. ELISA plate wells were coated with ECM proteins and further incubated with PDGF-BB and IL-1Ra variants at increasing concentrations. Graphs show signals given by antibodies detecting PDGF-BB or IL-1Ra. The signals were fitted by non-linear regression to obtain the dissociation constant (K_D) using A450 nm = B_{max}*[protein]/(K_D + [protein]) where [protein] is the concentration of PDGF-BB or IL-1Ra variants. Representative binding curves are shown. Data are means ± SD.

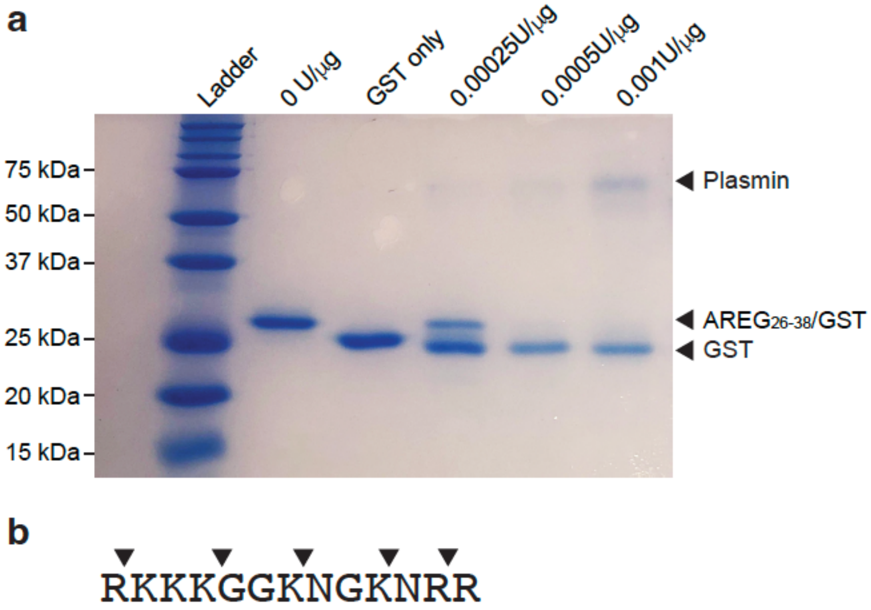

Supplementary Fig. 6. Plasmin cleavage of AREG$_{26-38}$. a AREG$_{26-38}$/GST was incubated with plasmin at increasing concentrations and further analysed by SDS-PAGE. The representative SDS-PAGE shows that AREG$_{26-38}$ is cleaved off GST by plasmin. Protein ladder is on the left. **b** Potential plasmin cleavage sites in AREG$_{26-38}$ indicated with black arrows.

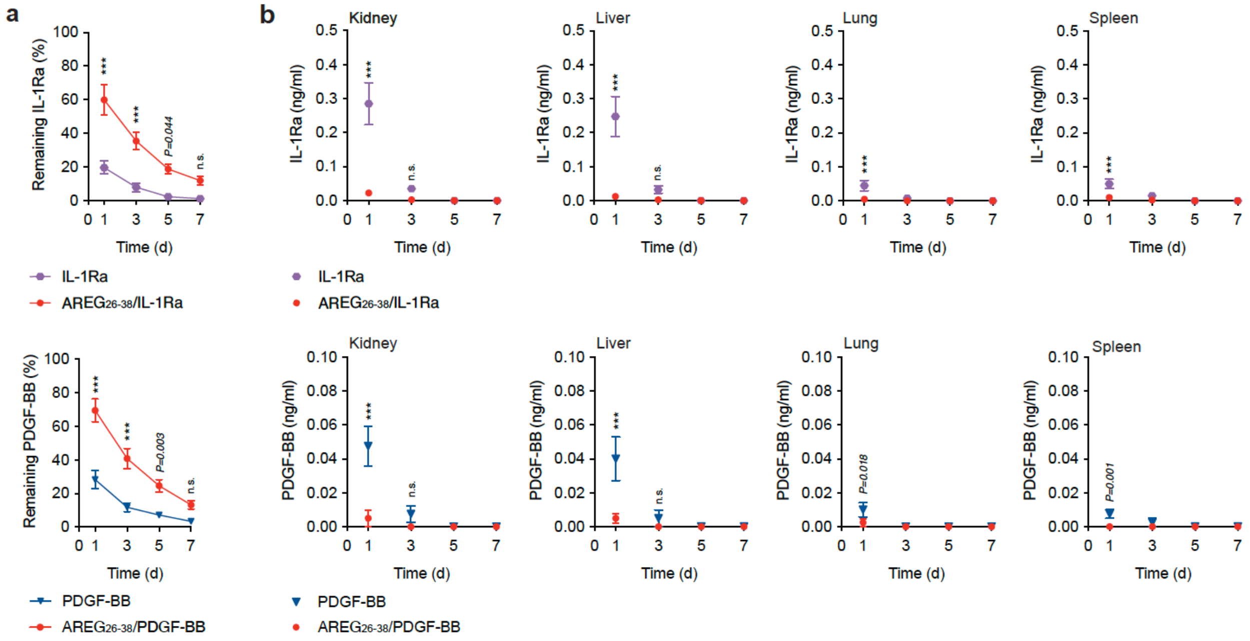

Supplementary Fig. 7. Retention and distribution of AREG$_{26\text{-}38}$-fused proteins after delivery in injured tissues. PDGF-BB variants were delivered via a fibrin matrix in muscle defects. IL-1Ra variants were injected in the skin surrounding full-thickness wounds. **a** Local retention of PDGF-BB and IL-1Ra. Graphs show the percentage of PDGF-BB and IL-1Ra variants remaining at the site of injection at various time points. $n = 4$ per time point. **b** Concentrations of AREG$_{26\text{-}38}$-fused proteins in various tissues following delivery. $n = 4$ per time point. For both panels, data are means ± SEM. Two-way ANOVA with Bonferroni *post hoc* test for pair-wise comparisons. $***P < 0.001$, otherwise indicated. n.s., non-significant.

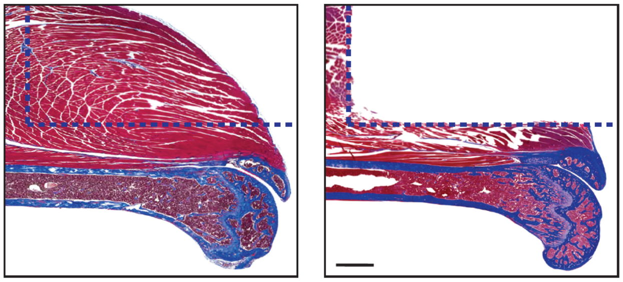

Supplementary Fig. 8. Histology of uninjured muscle and injured muscle immediately after injury. Representative histological sections with Masson's trichome staining of the centre of an uninjured leg (left) and injured leg (right) immediately after surgery. Muscle tissue is stained in dark red the femur stained in blue appears under the muscle. The dashed blue lines indicate the area of muscle removed. Scale bar, 1 mm.

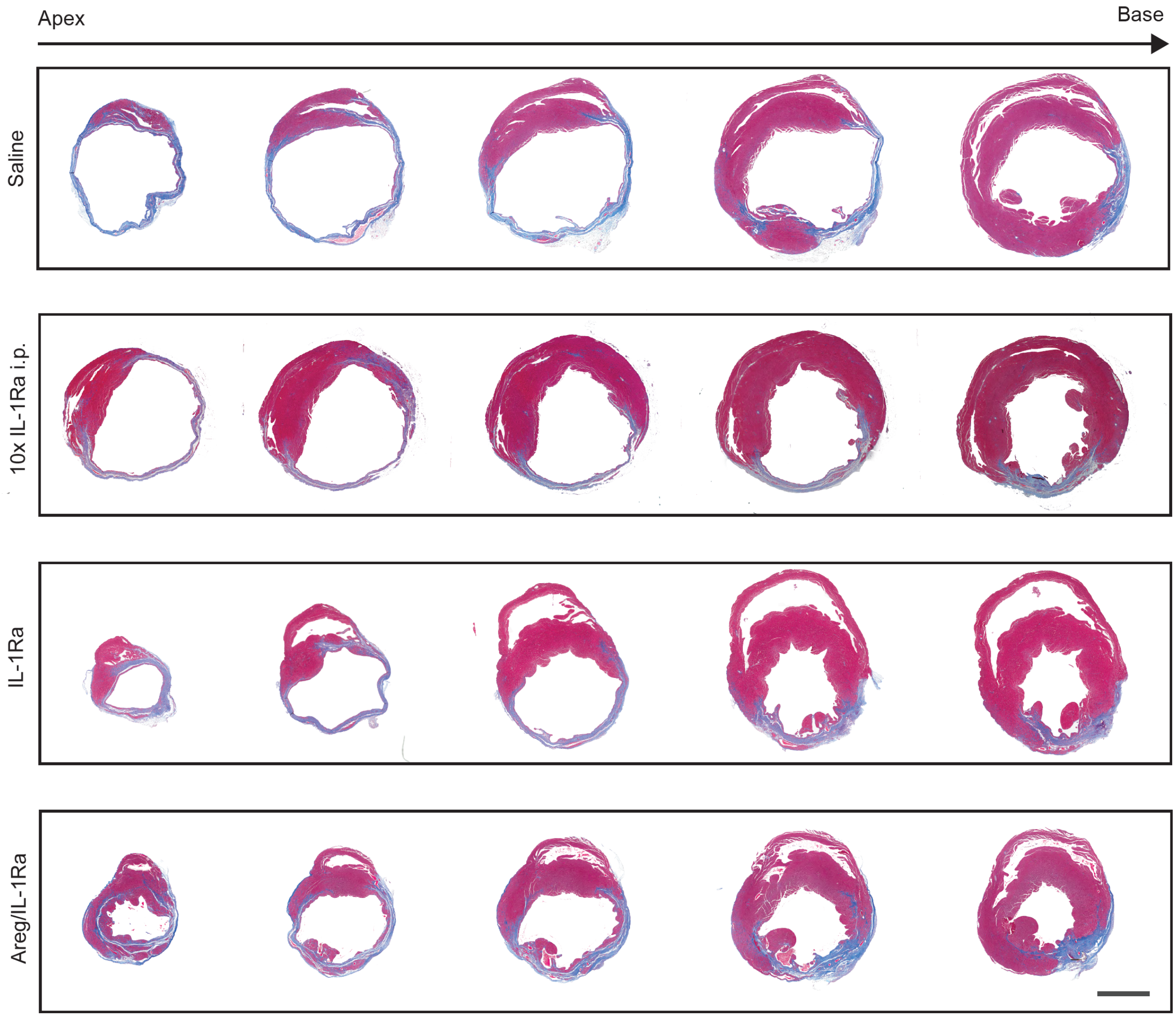

Supplementary Fig. 9. Intramyocardial delivery of $AREG_{26\text{-}38}$/IL-1Ra following myocardial infarction limits fibrosis. Permanent left coronary artery ligation was performed in mice to induce a myocardial infarct. IL-1Ra was delivered interperitoneally (40 μg) the day of the surgery or the borders of the infarct regions were injected with saline or IL-1Ra variants (4 μg of wild-type, equimolar $AREG_{26\text{-}38}$/IL-1Ra). *n* = 7. Representative histology (Masson's trichrome) 28 d post-treatment from the apex to the base of the heart. Healthy cardiac tissue appears in red and fibrotic tissue appears in blue/purple. Scale bar, 2 mm.

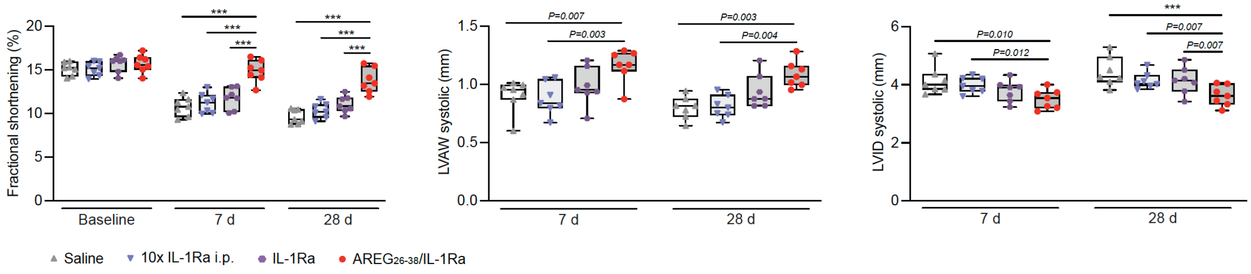

Supplementary Fig. 10. Intramyocardial delivery of $AREG_{26\text{-}38}$/IL-1Ra following myocardial infarction improves long term heart function. Permanent left coronary artery ligation was performed in mice to induce a myocardial infarct. IL-1Ra was delivered interperitoneally (40 μg) the day of the surgery or the borders of the infarct regions were injected with saline or IL-1Ra variants (4 μg of wild-type, equimolar $AREG_{26\text{-}38}$/IL-1Ra). *n* = 7. Left ventricular fractional shortening, left ventricular anterior wall (LVAW) systolic thickness (mm), and left ventricular internal diameter (LVID) in systole were measured at day 7 and 28 post-infraction. Data are plotted in box plots, box shows the median (centre line) and IQR (bounds). Whiskers show the minimum/maximum range. Two-way ANOVA with Bonferroni *post hoc* test for pair-wise comparisons was used. ***$P \leq 0.001$, otherwise indicated.

Supplementary Movie legend

Supplementary Video 1-4. Echocardiography of the heart four weeks post-treatment. Permanent left coronary artery ligation was performed in mice to induce a myocardial infarct. IL-1Ra was delivered interperitoneally (40 μg) the day of the surgery or the borders of the infarct regions were injected with saline or IL-1Ra variants (4 μg of wild-type, equimolar $AREG_{26-38}$/IL-1Ra). Cardiac function was assessed by echocardiography. The movie shows a left parasternal long-axis view in B-mode.

Unprocessed Western Blots

PDGFRα

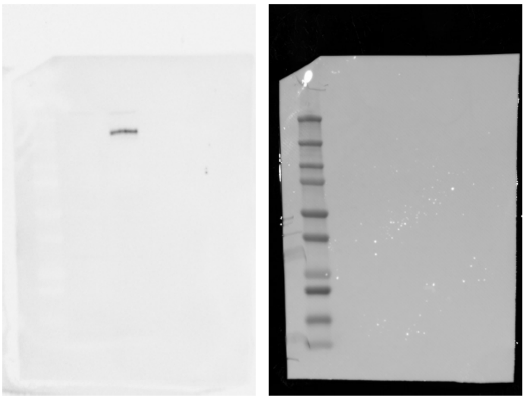

PDGFRβ

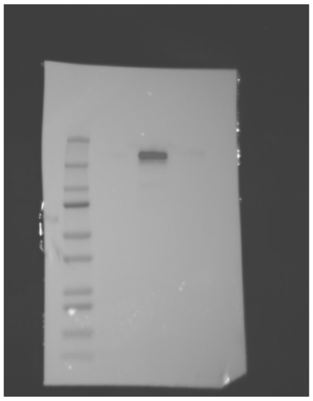

ACTIN (loading control)

PDGFRα blot PDGFRβ blot

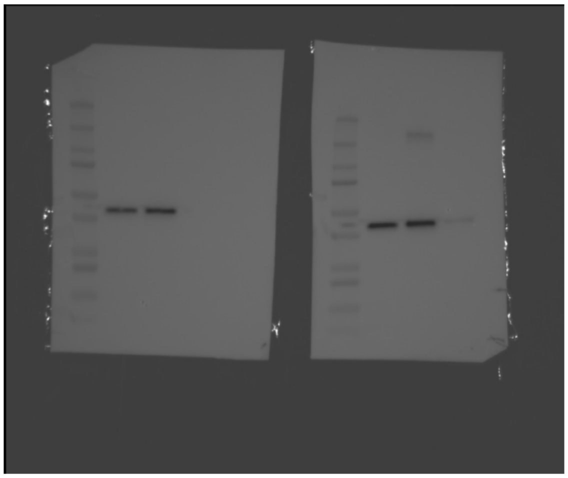

Supplement: Supplementary file 1 — SUPPLEMENTAL MATERIAL [file 41536_2023_297_MOESM1_ESM.pdf]
